# Engineering scalable vascularized kidney organoids for in vivo glomerular filtration with human endothelial integration

**DOI:** 10.1038/s44385-025-00063-5

**Published:** 2026-02-02

**Authors:** Murat Tekguc, Takuya Matsumoto, Lukas M. Altenburger, Kenichi Kobayashi, Ken Hiratsuka, Yuhei Higashi, Astia Rizki-Safitri, Tomoya Miyoshi, Wassim El-Jouni, M. Amin Arnaout, Thorsten R. Mempel, Ryuji Morizane

**Affiliations:** 1 Nephrology Division, Massachusetts General Hospital, Boston, MA USA; 2 Department of Medicine, Harvard Medical School, Boston, MA USA; 3 The Wyss Institute, Harvard University, Cambridge, MA USA; 4 Department of Medicine, Brigham and Women’s Hospital, Boston, MA USA; 5 Center for Immunology and Inflammatory Diseases, Division of Rheumatology, Allergy and Immunology, Massachusetts General Hospital, Boston, MA USA; 6 Broad Institute of MIT and Harvard, Cambridge, MA USA; 7 Harvard Stem Cell Institute, Cambridge, MA USA

**Keywords:** Biological techniques, Biotechnology, Cell biology, Engineering

## Abstract

Kidney organoids derived from human pluripotent stem cells have emerged as promising models for studying kidney disease and therapeutic development. However, the lack of a scalable production system has limited their industrial applications in regenerative medicine. Here, we have developed a cost-effective mass-production method for manufacturing vascularized kidney organoids, which has improved production efficiency by more than 50 times compared to conventional culture systems. The incorporation of a dynamic culture environment in delta-wing stirred bioreactors has significantly enhanced the glomerular vascularization of kidney organoids via mechanosensory integrin α2β1. Single-cell RNA sequencing and functional analyses demonstrated the enhanced maturation in STR nephron epithelia. The large quantities of vascularized kidney organoids enabled the fabrication of a nephron sheet with nephron numbers equivalent to those found in two rat kidneys. Intravital imaging of a nephron sheet implanted in a dorsal skinfold chamber of mice revealed filtration function with size selectability in the organoid glomeruli vascularized with human endothelia. This work may represent a significant step towards bridging the gap between basic research and commercial products, paving the way towards developing bioengineered kidneys for kidney replacement therapy.

## Introduction

Organoids are the three-dimensional (3D) aggregations of cells formed by a controlled self-organization program, representing tissue and organ structures in vitro^1,2^. The kidney, which consists of nephron structures of glomeruli and tubules, maintains body homeostasis by regulating fluid-electrolyte and acid-base balance via filtration of plasma and urinary excretion^3,4^. Kidney organoids have emerged as a novel model for studying kidney development, disease, and drug development. These organoids hold the potential to facilitate advancements in regenerative medicine for patients with kidney diseases^3–5^. As the rising number of patients with Chronic Kidney Disease (CKD) and End-Stage Kidney Disease (ESKD) underscores the urgent need for innovative renal replacement therapies, one potential solution to address this critical healthcare challenge is the development of implantable bioengineered kidneys derived from these organoids. Nevertheless, despite notable strides in refining the differentiation of human pluripotent stem cells (hPSCs) into kidney lineages, the practical large-scale production of functional kidney tissue is yet to be established.

Over the past decade, a plethora of directed differentiation protocols for generating kidney lineages from human pluripotent stem cells (hPSCs) have been documented^3,6–11^. Previous studies enabled the differentiation of hPSCs into nephron progenitor cells (NPCs) marked by the expression of critical markers such as SIX2, SALL1, WT1, and PAX2. These NPCs, in turn, possess the capacity to further develop into kidney organoids within the confines of 96-well culture plates. These organoids encompass vital components like podocyte clusters and renal tubular populations, comprising proximal tubules (PTs), loops of Henle (LOH), and distal tubules (DTs)^7,12^. However, this culture system presents certain challenges. Notably, the process involves time-consuming cell reseeding and media changes within the constraints of 96-well plates, which is labor-intensive. Additionally, the high costs associated with growth factors and ultra-low adhesion culture plates contribute to the financial barriers associated with this method. Consequently, these labor-intensive and cost-prohibitive experimental procedures hinder the scalability necessary for the mass production of kidney organoids, impeding the seamless transition from laboratory research to industrial applications, such as the future development of artificial kidneys.

It’s worth noting that previous conventional methods for generating kidney organoids rely on traditional static cell cultures, which limit the scalability of organoids in the absence of vascularized glomeruli, a pivotal structure responsible for the kidney’s blood filtration function^7,10,13^. Bioreactor-based dynamic flow culture systems were then introduced to increase the production capacity of organoids. However, differentiated organoids displayed the expansion of interstitial stromal cells and accumulation of collagen-rich extracellular matrix at a relatively early age, such as day 26^14^. Moreover, as the current state of technology is inadequate to support the ex vivo culture of human or even small animal kidneys, substantial advancements are required in bioreactor design to facilitate the cultivation of human-sized tissues, even if it becomes possible to create such large tissues in vitro. Here, we present a practical culture protocol using a stirred tank bioreactor (STR) with a delta-wing-shaped impeller designed to overcome the limitations of current kidney organoid production methods by boosting large-scale manufacture of kidney organoids exhibiting glomerular vascularization. This protocol aims to provide a labor- and cost-efficient, robust, and scalable platform for the mass production of functional vascularized kidney organoids that have the potential to be translated into current Good Manufacturing Practice (cGMP) compliant processes for the biofabrication of 3D kidney tissues^15^.

## Results

### 3D culture using stirred bioreactors enables the cost-efficient mass production of kidney organoids

**Fig. 1:**
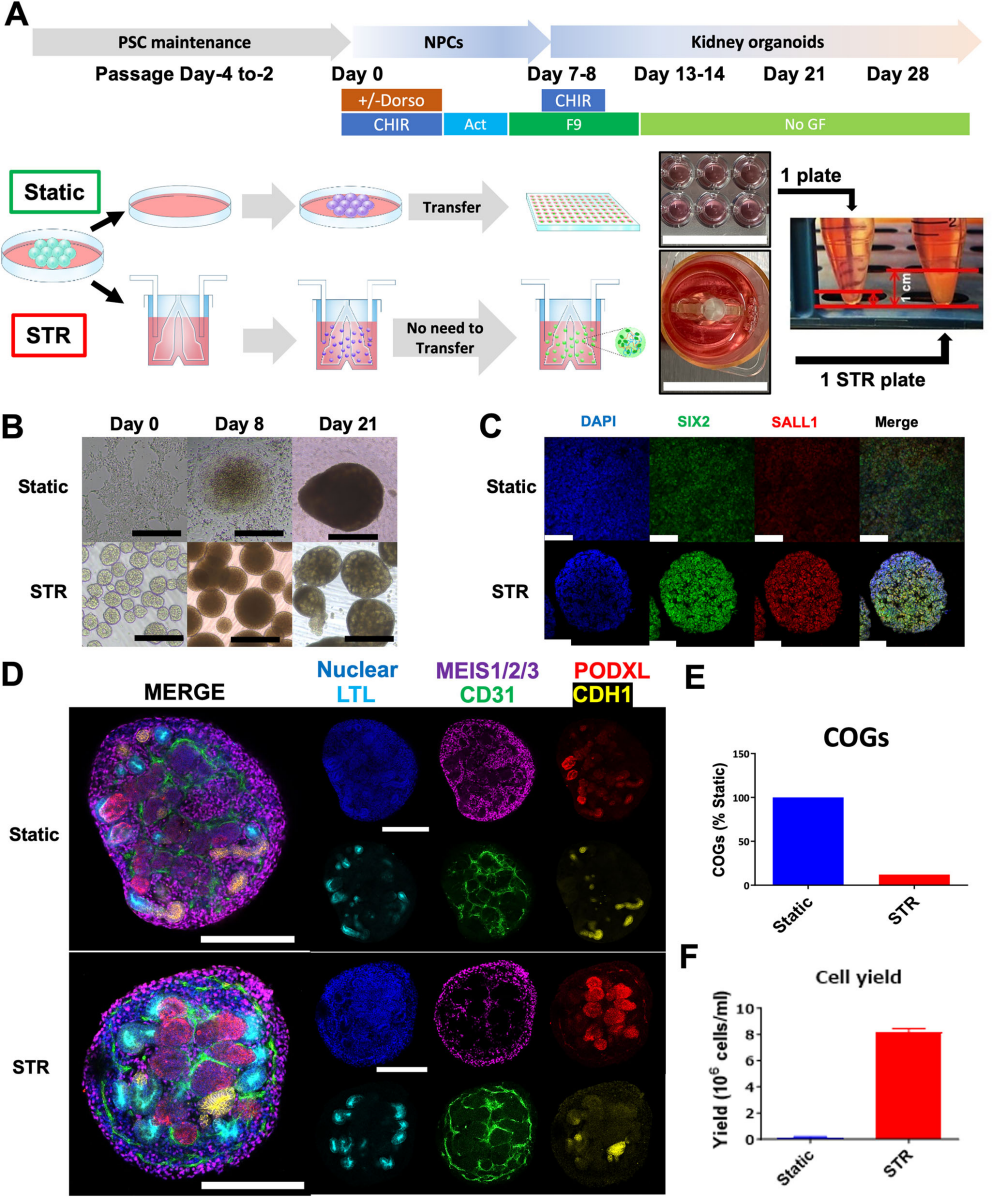
Establishment of a cost-efficient culture system for the mass production of kidney organoids. **A** Schematic procedures for kidney organoid differentiation from human PSC lines in static and STR culture systems. The schema explains the maintenance and differentiation process starting from PSCs to nephron progenitor cells (NPCs) and ultimately kidney organoids. STR and static organoids were treated with Activin for 2 or 3 days after 4 days of CHIR ± Dorsomorphin treatment. Spheroids were then treated with both CHIR and FGF9 starting on day 7 or 8 for 2 days, and subsequently, CHIR was removed. From day 13 or 14, no growth factors were added. The right-sided photo compares the final volumes of static (from 6 wells of one plate) and STR organoids, respectively. The scale bars: 25 mm. **B** Brightfield image of differentiating H9 cells in static and STR systems. The scale bar represents 500 µm. **C** Confocal images for the expression of nephron progenitor markers (SIX2 and SALL1) by H9 NPCs on day 8 of differentiation in static and STR systems. Scale bars: 100 µm. **D** Confocal images of day 21 STR-kidney organoids composed of proximal tubules (LTL: Lotus tetragonolobus lectin + ), interstitial cells (MEIS1/2/3 + ), endothelial cells (CD31 + ), podocytes (PODXL: Podocalyxin + ), loops of Henle/distal nephrons (CDH1: E-cadherin + ) with a scale bar of 200 µm. **E** Comparison of the total cost of goods/materials (COGs; cell culture reagents, media, and materials) in static and STR systems to generate the same cell number of kidney organoids. **F** Organoid cell numbers per media volume generated from one 96-well plate (static) and one 5-ml bioreactor (STR).

To streamline the culturing process, we devise a manufacturing process that can be carried out in a single STR using defined materials for large-scale production (Fig. 1A). Previous studies identified critical factors that require careful optimization for successful organoid differentiation in static culture^7,12^. These include cellular seeding densities and concentrations of CHIR and Dorsomorphin during the differentiation into primitive streak cells. Additionally, in this STR differentiation, we postulated that optimization is required for stirring rates and the duration of spheroid formation before initiating differentiation. The bioreactors were initially inoculated with a single cell suspension of undifferentiated hPSCs using both H9 female ES cells and BJFF.6 male iPS cells in a 5 ml culture media. To evaluate the quality of STR compared to static organoids, we performed detailed analyses such as 2D immunocytochemistry for nephron progenitor markers of SIX2 and SALL1 at the metanephric mesenchyme stage of differentiation on day 7–8, and whole-mount immunostaining of kidney organoids on day 21 to evaluate the compartments of differentiated nephron epithelia, stroma, and endothelia.

Nephron differentiation assessment by confocal microscopy and qPCR revealed that the spheroid size is a crucial parameter for the successful differentiation of kidney organoids in STRs. The spheroid size was determined by three parameters: (i) stirring rates, (ii) cell seeding densities, and (iii) the duration of maintenance culture in STRs before differentiation. The agitating rate of 80 revolutions per minute (rpm) was optimal to form spheroids in the 5-ml STRs in hPSC maintenance media, while faster or slower speed resulted in failure of spheroid formation or cellular adhesion to the bottom and side walls of STRs (Supplementary Fig. 1A). The spheroid size was correlated with higher seeding densities and longer culture duration (Supplementary Fig. 1B–F), and we found that the optimal diameter range is 100–200 μm to initiate the differentiation.

The induction of late mid-primitive streak for each hPSC line is a critical first step in the differentiation of metanephric kidney organoids^7^. To determine the optimal treatment for primitive streak induction, we conducted extensive experiments over the last four years using H9 and BJFF.6 hPSC lines with four independent researchers. During this extended period with multiple operators, the batch number of CHIR and dorsomorphin products changed several times. Specifically, we observed that CHIR concentrations must be adjusted based on the expression of SIX2 on days 7–8 and nephron number/morphology after day 21. For BJFF.6 cells, we found that dorsomorphin is also required. Furthermore, the different batches of CHIR often resulted in changes in differentiation outcomes, requiring re-optimization of its concentrations for each hPSC line (Supplementary Figs. 2, and 3A, B).

After optimizing the conditions for differentiation, this STR protocol enables highly efficient differentiation of H9 and BJFF.6 hPSCs into SIX2+ nephron progenitor cells within 7-8 days of induction (Fig. 1B, C). By transiently stimulating WNT signals using low-dose pulse CHIR treatment together with FGF9 for 2 days, the induced NPCs further differentiate into kidney organoids comprising multi-segmented nephrons. Multiplexed whole-mount immunostaining revealed the presence of distinct cell types, including PODXL + NPHS1 + NPHS2 + podocytes, LTL+ proximal tubules, CDH1+ loops of Henle/distal nephrons, MEIS1/2/3 + PDGFRβ+ stromal cells, and CD31+ endothelia (Fig. 1D and Supplementary Fig. 3C). In a side-by-side comparison between static and STR organoids by whole-mount immunostaining, we find similarities in nephron and interstitial cell induction (Fig. 1D and Supplementary Fig. 3C). However, the STR method exhibited superiority in organoid differentiation, cost, and yield of total organoid volume per batch (Figs. 1E, F, and Supplementary Fig. 4A, B). The STR system allows a 51-fold higher cell density in culture media than the static system (8.19 × 10^6^ cells/ml vs 1.59 × 10^5^ cells/ml), resulting in a significant cost reduction of 88%. Moreover, the total cell number from one STR is 13.1 times higher (4.2 × 10^7^ cells) than that from one 96-well plate (3.2 × 10^6^ cells), and the estimated labor required for the same total volume of organoids is reduced by 74% (Supplementary Fig. 4C–E). Our findings demonstrate the practical advantages of using the STR system for generating kidney organoids, which may enable the production of human-sized kidney tissues.

### Long-term culture of STR organoids

**Fig. 2:**
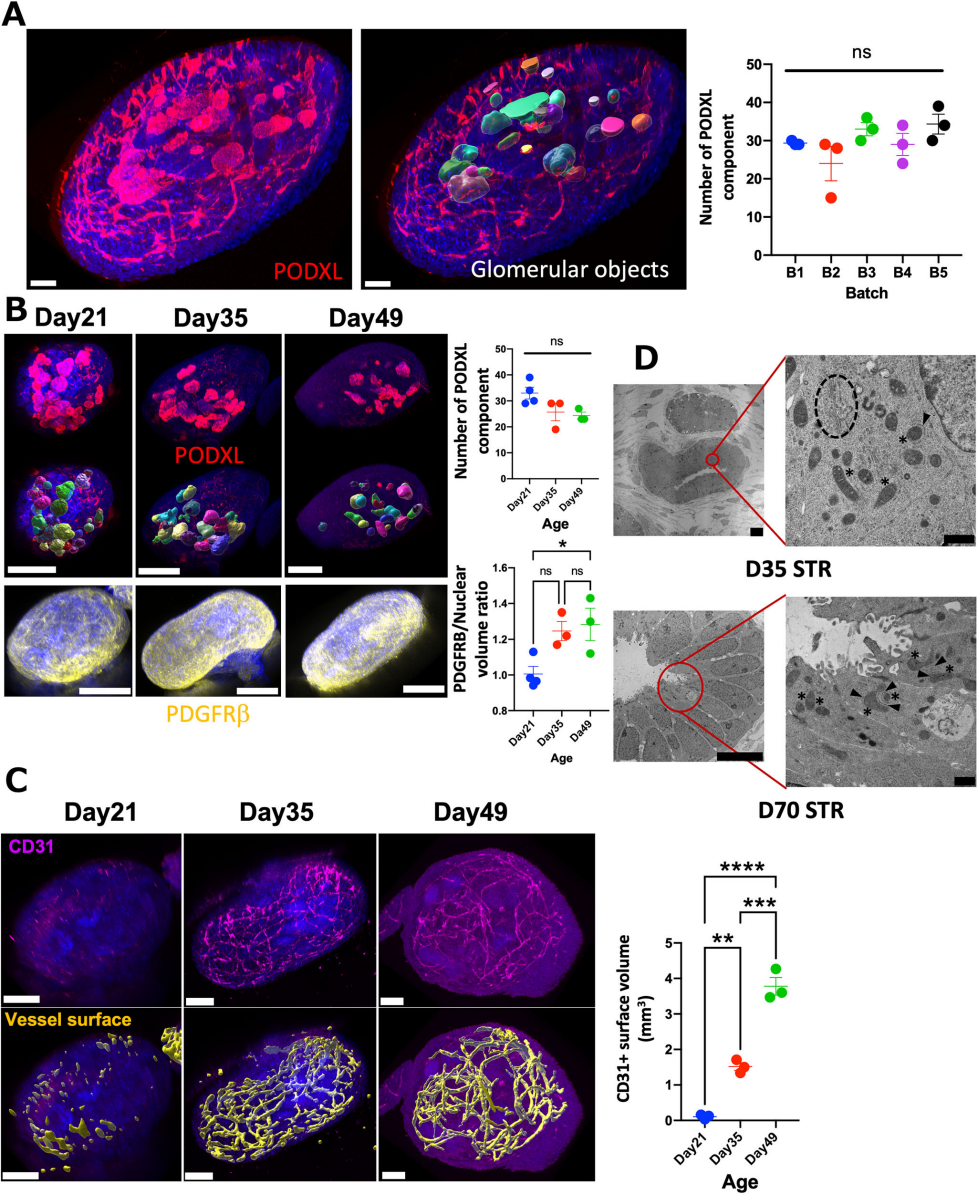
Long-term culture of kidney organoids in STRs. **A** 3D image reconstruction of glomerular surfaces in day 21 STR organoids. PODXL + 3D glomerular surface was generated via 3D surface rendering of Z-stack images by IMARIS Labkit segmentation and machine learning analysis. *n* = 15 organoids from 5 independent batches (3 organoids in each batch). Scale bars represent 50 μm. **B** Glomerular and stromal surfaces of STR organoids in time-course experiments. 3D surface rendering by IMARIS displays PODXL+ podocyte/glomerular and PDGFRβ + stromal surfaces in a time-course manner. The lower graph displays the ratio of PDGFRβ + surface volume to its nuclear counterpart. **C** The vascular surface of STR organoids in time-course experiments. 3D surface rendering by IMARIS displays CD31+ vascular surfaces in a time-course manner. Scale bars in (**B**, **C**) represent 200 and 100 μm, respectively. **D** Representative electron microscopy images of proximal tubular regions of STR organoids on days 35 and 70. The asterisks show multiple Mitochondria with their evident cristae structures, while the arrowheads point out Endoplasmic reticulum networks enveloping and interacting with adjacent Mitochondria in the metabolically active tubular epithelial cells. The black dashed line encircles the Golgi apparatus. The scale bars of the left low-magnification images represent 10 μm, while the scale bars of the right high-magnification images display 800 μm, respectively. Asterisks in the bar graphs (**A**–**C**) indicate *p* values derived from one-way ANOVA with Tukey’s multiple comparisons tests. Means ± SEM. (*p ≤ 0.05, **p ≤ 0.01, ***p ≤ 0.001, ****p ≤ 0.0001); ns, not significant.

The batch-to-batch variation of organoid differentiation is one major challenge in stem cell research^16^, and accurate quantitative measures of 3D structures are crucial to evaluate such variations. We utilized advanced 3D imaging based on a machine-learning approach using LABKIT, an ImageJ plugin^17^. The z-stack images of whole kidney organoids after tissue clearing were acquired with a confocal microscope to assess PODXL+ podocyte clusters, which are presumptive glomeruli on day 21 of differentiation^18^. While PODXL is also expressed in some endothelia^19,20^, this machine learning-based approach distinguished glomerular structures from vessels, enabling an accurate count of glomerular numbers in each organoid in multiple independent differentiation batches (Fig. 2A and Supplementary Fig. 3D, E). Each STR organoid contains approximately 30 podocyte clusters as pre-glomerular structures, and no statistical significance is observed in the number of podocyte clusters across these 5 batches.

To evaluate the stability of STR organoids in long-term culture, we then assessed the glomerular and stromal changes during 49 days of culture using the machine-learning approach. Our results showed a slight increase in PDGFRβ+ stromal volume from day 21 to 35, but overall, the number of PODXL+ glomeruli and PDGFRβ+ stromal volume remained similar until day 49 (Fig. 2B). Interestingly, vascular structures developed abundantly during the long-term culture (Fig. 2C). Furthermore, transmission electron microscopy analysis of STR organoids at early (D35) and late (D70) time point revealed tubular epithelial cells that developed brush border-like structures containing abundant organelles of well-defined endoplasmic reticulum, golgi apparatus, and basally- or apically-located mitochondria. These findings are indicative of a healthy and metabolically active status, without apparent evidence of tissue degeneration, even after 70 days of STR differentiation (Fig. 2D, Supplementary Fig. 5E left image). In contrast to previous studies in static culture, TEM images of STR organoids also pointed out the mitochondria-rich basal portions in the tubular epithelium, which are required for ATP-driven transporters^21,22^ (Supplementary Fig. 5E left image). These assessments by advanced 3D imaging highlight the long-term stability of STR organoids and their potential use in disease modeling and bioengineering studies.

### Transcriptomic evaluation by RNA-seq in static and STR organoids

**Fig. 3:**
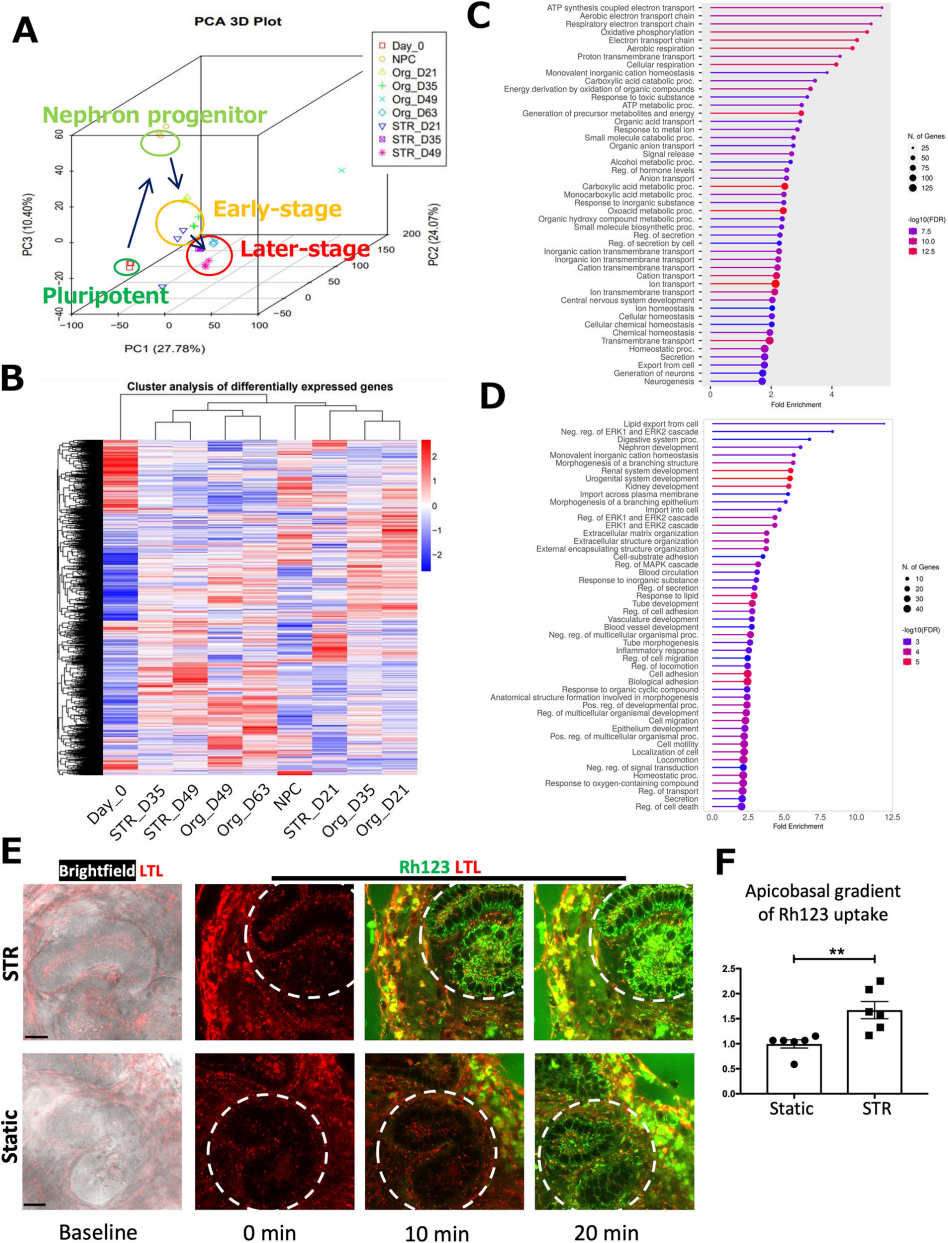
Comparative analysis and functional validation of gene expression profiles between STR and static kidney organoids. **A-D** Principal component (**A**) and hierarchical clustering (**B**) analyses from RNA-seq samples on day 0, day 8 (NPC), static day 21, 35, 49, 63 (Org_21, Org_35, Org_49, Org_63), and STR day 21, 35, 49 (STR_21, STR_35, STR_49). *n* = 3 mRNA samples. Each mRNA sample was collected from 6-12 organoids. Gene ontology (GO) analysis of DEGs significantly upregulated in STR organoids compared to static ones on day 21 (**C**) and day 35 (**D**). **E** Live-cell imaging in STR and static organoids to assess the transepithelial cation transport using Rhodamine 123 (Rh123). On days 32–38 of differentiation, both STR and static ones were transferred to the 1% Geltrex-coated 8-well glass-bottom chamber slides. On days 35–39, proximal tubules of organoids in these slides were labeled with LTL conjugated with Alexa Fluor 647. On days 36–40, after the organoids in chamber slides were assigned and mapped by the confocal microscope, the cation Rh123 (10 mM) was added into the culture media at a final concentration of 10 μM. Live-cell imaging was immediately started to check the efficiency of cation transport through LTL+ proximal tubular epithelial cells. Baseline brightfield images show the particular LTL+ proximal tubules before the addition of Rh123. Following the supplementation of Rh123, its uptake by tubular epithelial cells was recorded for 20–30 min via real-time imaging. The images encircled by white dashed lines exhibit the course of Rh123 uptake by LTL+ tubular epithelial cells at 0, 10, and 20 min. **F** Quantification of apical and basal Rh123 fluorescence intensities of proximal tubular epithelial cells to calculate the apicobasal ratio of Rh123 uptake for the assessment of cation transport from basal to apical sides in these tubular epithelial cells. Each dot represents the proximal tubular histogram analysis of each organoid, *n* = 6 organoids from 2 independent organoid batches of BJFF.6 and HUES62 cell lines. The asterisk in the bar graph indicates the *p* value derived from a two-tailed unpaired t-test. Means ± SEM. **p ≤ 0.01.

To assess global gene expression profiles in STR and static kidney organoids, we performed next-generation sequencing (RNA-seq) from static samples collected on days 0, 8 (NPC), 21, 35, 49, and 63 and compared them to those from STRs on days 21, 35, and 49. Principal Component Analysis (PCA) showed a time-dependent change of global gene expression profiles from undifferentiated hPSCs (day 0) to late-stage kidney organoids (day 63) (Fig. 3A). Furthermore, hierarchical clustering demonstrated similarities between day 21 STR organoids and day 21/35 static organoids, while day 35/49 STR organoids were more closely related to day 49/63 static organoids (Fig. 3B). Taken together, these findings indicate that gene expression changes occur over time in both STR and static kidney organoids, but the longitudinal maturation process appears to be accelerated in STR organoids compared to static kidney organoids.

To investigate the differences between STR and static organoids, we conducted differential gene expression (DEG) analyses comparing the two types of organoids at equivalent stages of differentiation. Our analyses revealed the upregulation of 1225 genes in STR organoids on day 21 and 588 genes on day 35. To gain insight into the biological processes associated with these upregulated genes, we performed gene ontology (GO) analyses. By assessing the Fold Enrichment values, we visualized the top 50 GO terms using ShinyGO (Fig. 3C, D)^23^. Notably, the upregulated genes in STR organoids on day 21 showed strong associations with transporter activities such as “Organic anion transport” and “Cation transport”. Further, the samples from day 35 exhibited an increased association of STR organoids with “Kidney Development” and “Vasculature Development”.

To evaluate the similarities in nephron differentiation between the STR and static organoids, we examined the expression levels of commonly used markers for each segment of nephron epithelia, such as MAFB, SLC34A1, and SLC12A1. Our analysis revealed that the expression levels of nephron segment markers, including Podocyte, PT (proximal tubule), and LOH/DT (loop of Henle/distal tubule), were comparable in both organoids (Supplementary Fig. 4F). Additionally, we observed similar expression patterns for certain drug and albumin transporter genes in both types of organoids, though some were higher in STR organoids. Of note, the expression of muscle genes was significantly downregulated in STR organoids, while the expression levels of neural genes were comparable between the two.

Based on the increased expression of organic cation transporter-2 (SLC22A2) and multidrug and toxin extrusion protein 1 (SLC47A1) in STR organoids, we performed a functional live-cell imaging assay to visualize cation transport in LTL+ proximal tubules. Organoids were generated in STR, and their static control samples were transferred from STR to the static culture condition on day 14 of differentiation. Proximal tubules were visualized with fluorescent-conjugated LTL in live organoids on day 40 of differentiation, and Rhodamine 123 (Rh123), a cationic fluorescent dye, was added to the culture media as previously described^24^. While both static and STR organoids exhibited Rh123 uptake and transport to the lumens of LTL+ tubules, the apical transport of cationic Rh123 from basal sides of tubules was significantly enhanced in STR organoids during the live-cell imaging. The apicobasal ratios of Rh123 signals were significantly higher in STR organoids, suggesting that they enhanced cation transport compared to static counterparts (Fig. 3E, F, and Supplementary Fig. 4G).

These assessments of global gene expression by RNA-seq and the functional assay suggest an improved maturation and vascularization process and reduced off-target differentiation in kidney organoids generated using the STR method.

### Glomerular vascular development is enhanced in STR organoids

**Fig. 4:**
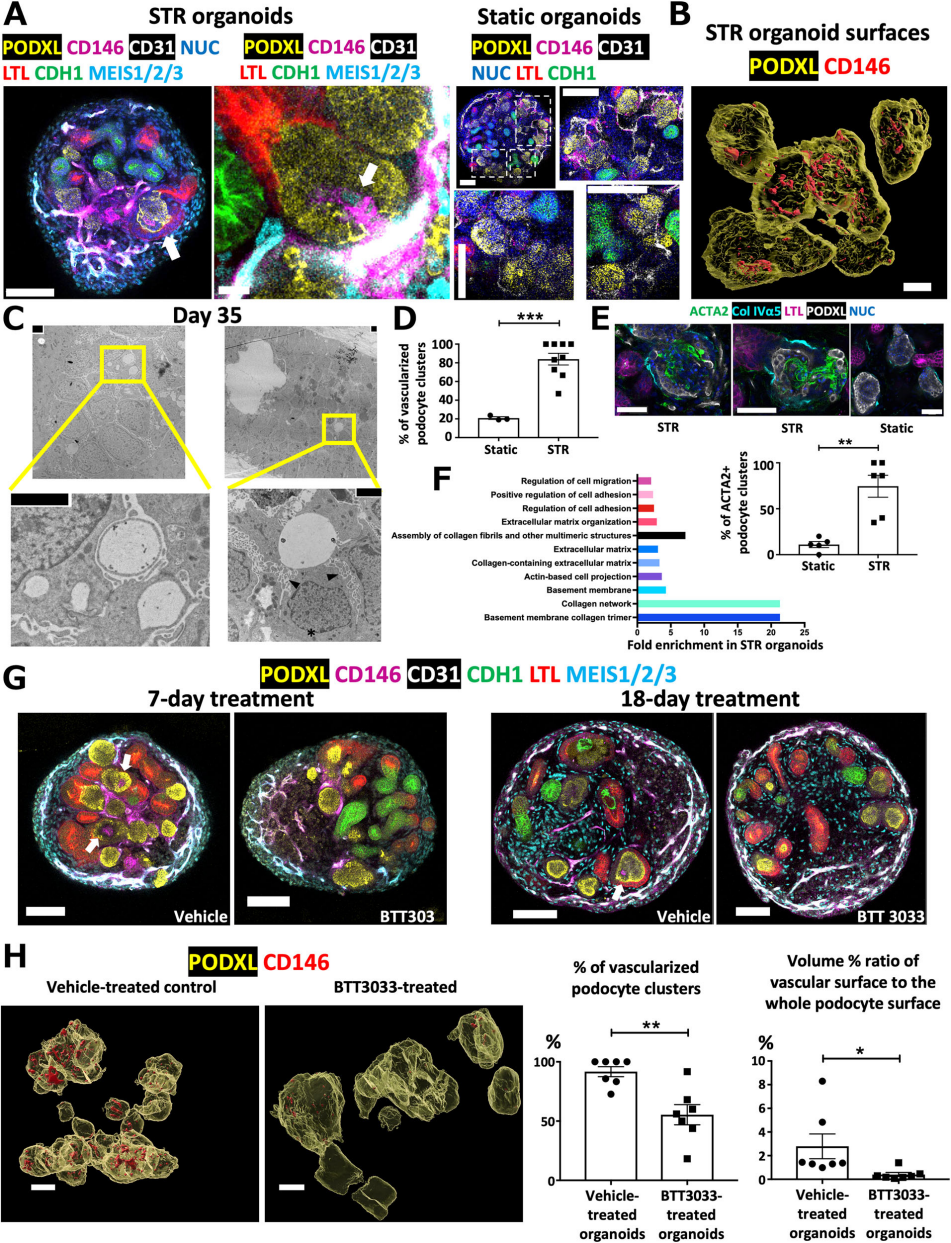
In vitro glomerular vessel development in STR organoids with human endothelial and stromal cells. **A** Vessel sprouts in polarized podocyte clusters with CD146+ capillary network supported by MEIS1/2/3+ stromal cells in day 21 STR organoids (left set of two images) compared to the podocyte clusters of day 21 control static organoids (right set of four images). White arrows in the STR images indicate the vascularized glomeruli surrounded by polarized podocytes. Scale bars of STR images represent 100 μm and 10 μm (zoomed STR image), respectively. Rectangular white dashed lines point out the zoomed images of non-vascularized podocyte clusters of the static organoid. Scale bars of static organoid images represent 100 μm. **B** 3D surface reconstruction of CD146+ capillary network and surrounding PODXL+ podocyte clusters as vascularized glomerular components. Z-stack images were acquired at a step size of 2 μm. The scale bar represents 30 μm. **C** Transmission electron microscopy images show multiple vascular lumens (left image) and accompanying podocyte foot processes around the vascular lumen (right image) within organoid glomerular structures on day 35. The asterisk indicates the nucleus of the podocyte surrounding the vascular lumen, while the arrowheads mark podocyte foot processes. Black scale bars at the corners: 2 μm. **D** Percentages of vascularized podocyte clusters containing CD146 endothelial surface in STR versus static organoids via 3D surface reconstruction by machine learning. *n* = 3 static and *n* = 9 STR organoids from 3 independent batches differentiated from BJFF.6 or HUES62 cells. **E** ACTA2+ mesangial-like cells surrounded by polarized PODXL+ podocytes in Bowman’s space of day 35 STR and static organoids. STR organoids were generated by the transfer of the same-batch static organoids into STR wells on day 14 of differentiation. The graph represents the percentage of organoids containing ACTA2+ cells (*n* = 5 static and n = 6 STR organoids from 2 independent batches differentiated from H9 or HUES62 cells). **F** Upregulation of certain GO terms associated with extracellular matrix organization, cell adhesion, and migration in day 35 STR organoids compared to static organoids. “Regulation of cell migration”, “Positive regulation of cell adhesion”, “Regulation of cell adhesion”, and “Extracellular matrix organization” terms constitute GO biological processes while “Extracellular matrix”, “Collagen-containing extracellular matrix”, “Actin-based cell projection”, and “Basement membrane”, “Collagen network”, and “Basement membrane collagen trimer” terms can be classified as GO cellular components, and “Assembly of collagen fibrils and other multimeric structures” is a part of Reactome pathways. **G** Inhibition of glomerular vascularization of STR organoids by the application of α2β1 integrin inhibitor, BTT3033 (stock concentration of 10 mM), with a final concentration of 1 μM for 7 or 18 days, in contrast to the control vehicle-treated organoids. The white arrows in the confocal microscopy images indicate the vascularized glomeruli surrounded by polarized podocytes. The scale bars represent 100 μm. **H** 3D surface reconstruction of CD146+ capillary network and surrounding PODXL+ podocyte clusters as vascularized glomerular surfaces in vehicle- and BTT3033-treated organoids. Z-stack images were acquired at a step size of 2 μm. The scale bar represents 50 μm. Y-axes of graphs represent two parameters: the percentage of vascularized podocyte clusters and the volume percent ratio of CD146+ vascular surface in these podocyte clusters to the whole PODXL+ podocyte surface, respectively (*n* = 7). Asterisks in the bar graphs (**D**, **E**, **H**) indicate *p* values derived from two-tailed unpaired t-tests. Means ± SEM. (*p ≤ 0.05, **p ≤ 0.01, ***p ≤ 0.001).

Glomerular vascularization is crucial for kidney function as it enables urine production through blood filtration. However, static kidney organoids do not naturally develop vascular capillaries within their glomeruli in vitro. Upon transplantation into animals, host vessels infiltrate organoid glomeruli, forming chimeric tissues that do not faithfully replicate human glomerular function and disease^25^. Building upon transcriptomic data hinting at vascular enhancements in STR organoids, we investigated glomerular vessel development using whole-mount immunostaining with tissue clearing. This approach revealed robust vessel formation throughout the interstitial space of STR organoids, marked by both CD146 and CD31 endothelial markers (Fig. 4A, left image). Of particular note, detailed z-stack imaging of individual glomeruli unveiled glomerular microvessel formation characterized by CD146+ endothelial cells extending from interstitial vascular structures and MEIS1/2/3+ mesangial-like stromal cells, all surrounded by PODXL+ podocytes (Fig. 4A, middle image, Supplementary Fig. 5A, B). Moreover, we detected the cellular polarization of podocytes through the concentration of PODXL at the apical surface, distinct from the compact non-polarized podocyte clusters of the control day 21 organoids maintained under static conditions (Fig. 4A and Supplementary Fig. 5B). CD146+ endothelial cells penetrated the internal sites of STR podocyte clusters together with MEIS1/2/3+ stromal cells (Fig. 4A, left and middle STR images, Supplementary Fig. 5A-C), whereas static podocyte clusters were circumferentially surrounded by these vessels without evident internal glomerular vascularization (Fig. 4A, right static images, Supplementary Fig. 5D). Furthermore, peritubular capillaries encircled proximal and distal tubular structures of STR organoids, similar to their physiological counterparts (Supplementary Fig. 5C). The unbiased assessment by 3D imaging using IMARIS software and machine learning confirmed that STR glomeruli (3D podocyte surfaces) exhibited enhanced vessel sprouts with CD146+ endothelia (Fig. 4B).

To assess the ultrastructure and extended durability of vascularized glomeruli in STR culture, we utilized electron microscopy on days 35 and 70 of STR differentiation. Day 35 organoid glomeruli exhibited multiple vascular lumens, resembling capillary loops (Fig. 4C left image). Additionally, the organoid podocytes maintained cellular polarization and surrounded the capillary-like structures, projecting their foot processes to the basement membranes of vascular endothelial cells (Fig. 4C right image, Supplementary Fig. 5E middle and right images). Quantitative assessments revealed a significant increase in the percentage of glomeruli containing CD146 endothelia (vascularized podocyte clusters) in STR organoids compared to their static counterparts, as well as a notably higher abundance of ACTA2+ presumptive mesangial cells in STR glomeruli (Fig. 4D, E, and Supplementary Fig. 5F).

Mechanical stress was considered the trigger for glomerular vascularization, in line with previous studies reporting vascularization induced by fluid flow in vitro or blood flow in vivo^26,27^. However, the precise molecular mechanisms governing such vascular invasion remain to be elucidated. Interestingly, we observed an upregulation of certain genes in STR organoids, which exhibited significant associations with downstream signaling patterns of integrins such as “Regulation of cell adhesion”, “Extracellular matrix organization”, “Regulation of cell migration”, “Collagen network”, “Assembly of collagen fibrils and other multimeric structures”, “Actin-based cell projection” in day 35 STR organoids as well as the expression of varied integrins in day 21 and 35 STR organoids compared to their static counterparts (Fig. 4F and Supplementary Fig. 5G). Integrins are transmembrane proteins responsible for the integration and reorganization of the extracellular matrix and cytoskeletal protein network^28,29^. They play crucial roles in mechanotransduction stimulated by mechanical stress^30^. Therefore, we hypothesized that integrins mediate glomerular vascularization in STR organoids during early nephron formation. Indeed, glomerular vascularization was similarly promoted in STR organoids even if these organoids were transferred from static to STR condition on day 14 of differentiation (Supplementary Fig. 5F). To test our hypothesis, we first checked the expression of integrin α2 protein, which appeared to be colocalized with PDGFRβ+ mesangial-like cells surrounding the glomerular capillary-like structures of STR organoids by immunostaining (Supplementary Fig. 5H). We then conducted perturbation experiments by introducing an integrin inhibitor, BTT3033, into the culture media from differentiation day 14 to days 21 and 32 (Fig. 4G). We selected this inhibitor for its specificity to integrin α2β1, whose subunits are co-expressed in human mesangial cells, while its β1 component can also be detected in glomerular capillary endothelia regardless of its α2 subunit, according to human single-cell data by the Kidney Precision Medicine Project^31^.

While BTT3033 appeared to have no substantial impact on interstitial vessel formation, we rarely observed vascular invasion into glomeruli with the inhibition of integrin α2β1 under 7- and 18-day treatment (Fig. 4G, H). Upon the 3D reconstruction of podocyte (PODXL + ) and vascular (CD146 + ) surfaces, we checked two parameters, percentage of vascularized podocyte clusters and volume percent ratio of vascular surface in podocyte clusters to the whole podocyte surface, to check the 3D changes in glomerular vascularization upon treatment (Fig. 4H). Both these 3D surface parameters and the immunostaining of glomerular endothelial marker (EHD3) demonstrated that integrin α2β1 inhibition significantly abolished glomerular vessel sprouting in BTT3033-treated STR organoids, in contrast to vehicle-applied controls (Fig. 4H and Supplementary Fig. 5I). Considering that external mechanical forces lead to the recruitment, activation, and assembly of downstream cytoskeletal scaffolds, such as integrin adhesion complexes^32,33^, our results suggest that the fluidic environment in STRs induces integrin expression and/or signals, thereby facilitating endothelial invasion into glomeruli. This finding is consistent with previous research that highlighted the role of mesangial integrins, required for functional mechanotransduction machinery^34^, in the glomerulogenesis, and development and maintenance of glomerular capillary integrity^28,35,36^. According to CellChat (a ligand-receptor database), integrin α2β1 interacts with Laminin 521, which is one of the components of glomerular basement membranes. Prior studies have shown reduced glomerular vessel formation in Laminin α5 knock-out models, consistent with our findings^37^.

### Single-cell RNA sequencing in STR organoids and their static counterparts

**Fig. 5:**
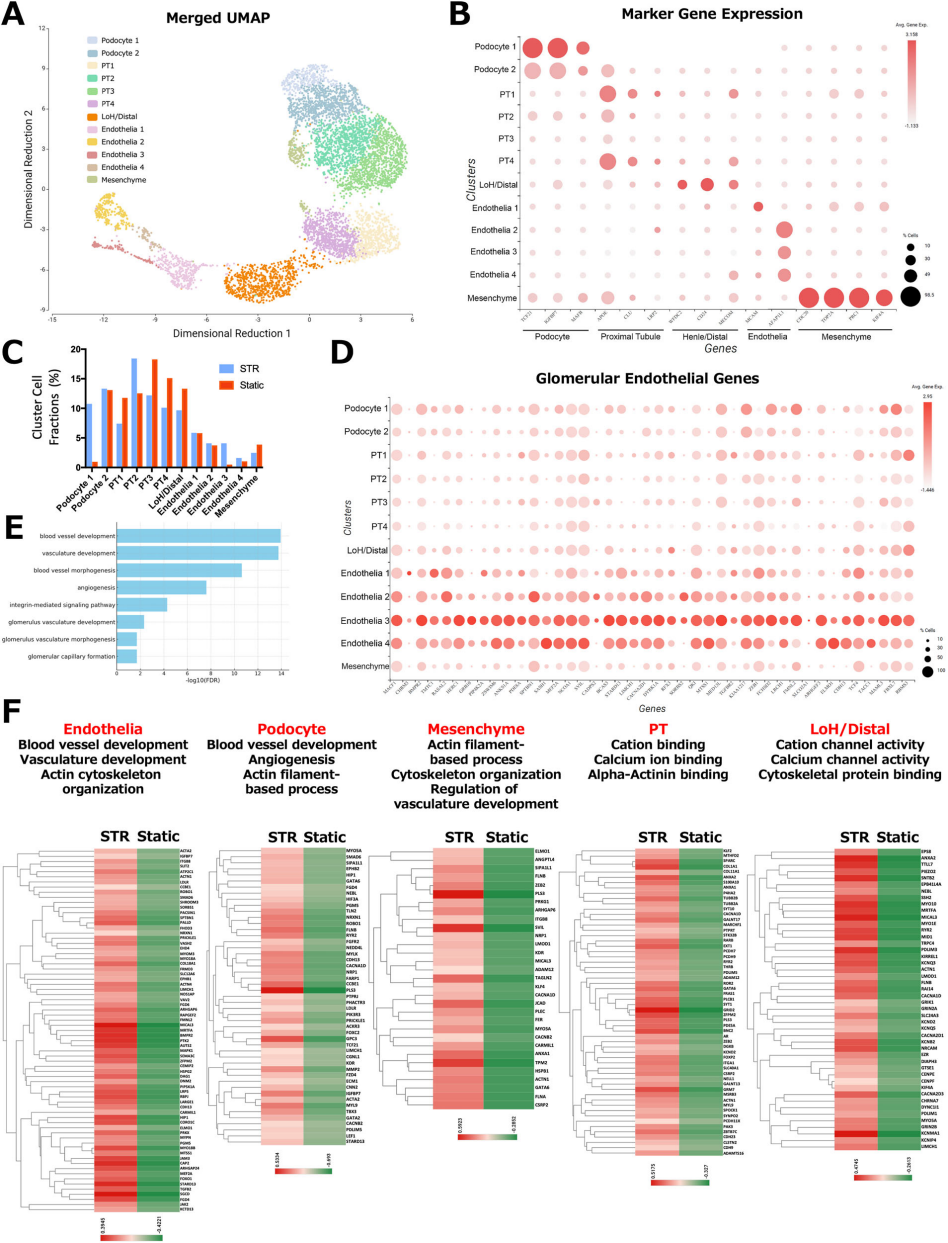
Single-cell RNA-seq analysis in STR organoids. **A** Visualization of Uniform Manifold Approximation and Projection (UMAP) from merged matrices of static (3476 cells) and STR (2633 cells) organoids on day 36 of differentiation. **B** Marker gene expression in each cluster from the merged UMAP of static and STR samples. **C** The graph shows the cellular fractions of each UMAP cluster in STR and static organoids. **D** The expression (42/137 genes) of human glomerular endothelial genes (GSEA: M39241, LAKE_ADULT_KIDNEY_C22_ENDOTHELIAL_CELLS_GLOMERULAR_CAPILLARIES) across all clusters in merged UMAP. **E** A bar graph showing representative GO terms of biological processes significantly associated with DEGs in the Endothelial 3 cluster when compared against all other clusters. **F** Heatmap charts display the DEGs corresponding to the particular GO terms of biological processes (Endothelia, Podocyte, Mesenchyme) and molecular functions (PT and LoH/Distal) in STR organoids compared to static ones. Each cluster represents the combined set of subclusters for the corresponding cell type.

Based on the finding of reduced CD146+ or EHD3+ glomerular endothelia by the integrin α2β1 inhibition from day 14 of differentiation, we next assessed the effects of dynamic culture environments of the stirred bioreactors using single-cell RNA sequencing (scRNA-seq). Kidney organoids were first generated in static culture and subsequently transferred to STR culture on day 14 of differentiation. Organoids were dissociated into single cells and underwent scRNA-seq analysis on day 36 of differentiation. After quality control filtering, 6109 cells (static: 3476; STR: 2633 cells) were visualized on Uniform Manifold Approximation and Projection (UMAP) with 9476 mean counts per cell using Seurat^38^. As previously described, cell-specific anchor genes were used to identify clusters, which included populations of endothelial cells (Endothelia), podocytes (Podocyte), mesenchymal cells (Mesenchyme), proximal tubular cells (PT), loop of Henle/distal tubular cells (LoH/Distal) (Fig. 5A, B)^39^. We found that 2 clusters, Podocyte 1 (STR 10.75% vs static 0.95%) and Endothelia 3 (STR 4.1% vs static 0.49%), showed higher percentages in STR organoids among all other clusters (Fig. 5C and Supplementary Fig. 6A, B). Differential expression gene (DEG) analysis in Endothelia 3 cluster against all other clusters revealed significantly upregulated expression of human glomerular endothelial genes and significant association with multiple biological processes, including blood vessel development, angiogenesis, integrin-mediated signal pathways, and glomerular vascular development (Fig. 5D, E). Subsequently, we assessed ligand perturbations using the upregulated DEGs in the Endothelia 3 cluster. Interestingly, this analysis suggested the involvement of estradiol, TGF-β1, insulin-like growth factor-1, calcitriol, and interleukins in glomerular vessel development (Supplementary Data 1). Further analysis of DEGs in Podocyte 1, the other cluster highly upregulated in STR organoids, also showed significant association with certain biological processes such as sprouting angiogenesis, endothelial cell migration, blood vessel development, and organization of cytoskeleton and cell projection, supporting the link between glomerular vascularization and integrin signaling (Supplementary Fig. 6C).

To assess the overall effect of STR culture on the molecular features, we performed DEG and GO term analysis in Endothelia, Podocyte, Mesenchyme, PT, and LoH/Distal clusters of STR organoids. A significant enhancement was found in biological processes associated with blood vessel and vasculature development and angiogenesis in STR clusters of Endothelia, Podocyte, and Mesenchyme. As expected, we also found significant upregulation in the downstream cascades of integrin-mediated mechanotransduction, such as actin cytoskeleton organization and actin filament-based processes (Fig. 5F and Supplementary Fig. 6C, Supplementary Data 2).

Additionally, consistent with previous bulk RNA-seq results (Fig. 3C), we found the upregulation of genes associated with cation and calcium ion binding, cation and calcium channel activities in STR PT and LoH/Distal clusters (Fig. 5F and Supplementary Fig. 6C), further supporting our result of the enhanced Rh123 cationic transport in the STR proximal tubules during live-cell imaging (Fig. 3E, F).

### Prevascularized nephron sheets perform filtration function when implanted into NSG mice through the anastomosis between the human endothelial network and host murine vasculature

**Fig. 6:**
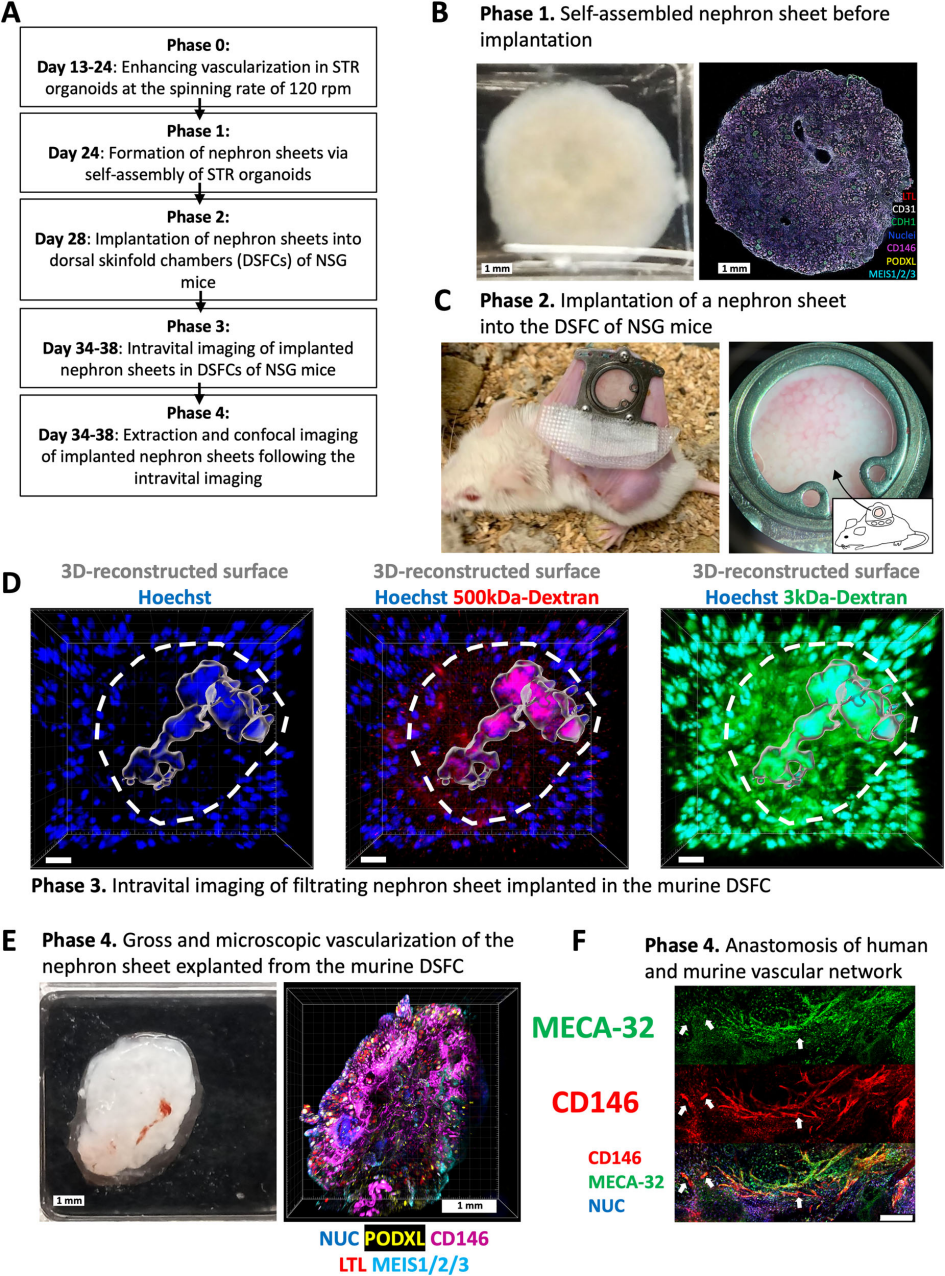
Implantation of pre-vascularized human nephron sheets into mice facilitates size-selective filtration of LMW dextran via graft-host anastomosis of human and murine capillaries. **A** Workflow for the implantation of nephron sheets generated from STR organoids into dorsal skinfold chambers (DSFCs) of NSG mice. Implantation of STR organoids into mice was performed six times from three independent organoid batches. **B** Phase 1 left photo shows the self-assembled nephron sheet formed by STR organoids. Instead of implantation, this sheet was fixed with 4% PFA on day 28 to confirm the self-assembly process. The right image represents the stained nephron sheet with the designated markers (LTL, CD31, CDH1, CD146, PODXL, MEIS1/2/3) following the fixation process. The scale bars represent 1 mm. **C** Phase 2 photos include the NSG mouse with a dorsal skin fold chamber (DSFC) on its back (left photo) and the implanted nephron sheet generated from day 24 STR organoids inside the DSFC (right photo). **D** Phase 3 multiphoton intravital microscopy (MP-IVM) of the implanted sheet reveals the nephron units (designated by a circular white dashed line) with the dynamic flow of both high-molecular-weight (HMW, 500 kDa-Cy5) and low-molecular-weight (LMW, 3 kDa-FITC) dextrans in glomerular vascular-like structures (designated by 3D-reconstructed gray surface), accompanied by the filtration of LMW dextran into the surrounding space. The 3D-reconstructed cellular surface was generated via 3D surface rendering of Hoechst in Z-stack images by IMARIS. The scale bar displays 20 μm. **E** Phase 4 left photo shows the gross vascularization of the implanted nephron sheet with murine vessels on the 6th day of implantation, following its extraction and fixation. 3D reconstructed image (right) demonstrates the ongoing CD146+ human vascular network inside the same phase 4 nephron sheet (PODXL, CD146, LTL, MEIS123). Z-stack images were acquired at a step size of 100 μm. The scale bars represent 1 mm. **F** The confocal microscopy image showing the anastomosis of MECA-32-CD146+ human (white arrows, single-positive red) and MECA-32+ murine vascular endothelial networks. The scale bar displays 150 μm.

In this study, our next step was to generate large nephron sheets that contain a nephron count comparable to that of small animals, serving as a proof-of-concept for the eventual creation of kidney organoids with sufficient volume for regenerative applications (Fig. 6A). To achieve the fabrication of these nephron sheets from STR organoids, we conducted experiments with various Extracellular Matrices (ECMs), among which fibrinogen, gelatin, or laminin511 had negative effects on nephron differentiation (Supplementary Fig. 7A). Matrigel extracted from mouse sarcomas did not show an apparent impact on nephron differentiation; however, such animal-derived products may pose obstacles to future transition to GMP-grade manufacturing. Consequently, we generated nephron sheets by simply inserting the collection of STR organoids in wide-bore tips (a volume range of 25–100 μl) onto a transwell plate without any exogenous ECM. Therefore, the formation of circular sheet-like structures was facilitated via self-fusion of such a substantial number of organoids solely based on the capacity of their endogenous ECMs (Fig. 6B). When the nephron sheet was prepared from 100 μl-volume of STR organoids, the number of nephrons (~30 nephrons/podocyte clusters per organoid) obtained from a 100-μl sheet (~1533 organoids) was calculated via machine learning in IMARIS as 46,000 nephrons, nearly twice size of a rat kidney with a range of 13,000 to 26,000 nephrons^40^ (Fig. 2A and Supplementary Table 3).

To assess the stability of organoid glomeruli vascularized with human endothelia after implantation into animals, we conducted implantation experiments using immunocompromised NSG mice. In previous studies, kidney organoids were typically transplanted into the renal subcapsular space, necessitating complex and invasive surgical procedures for both implantation and subsequent intravital imaging. Our approach aimed to minimize animal stress while enabling repetitive live-cell imaging. To achieve this, the nephron sheets were implanted into dorsal skinfold chambers (DSFCs) that were surgically installed on the back regions of NSG mice (Fig. 6C). This innovative method allowed us to monitor the organoid glomeruli in a less invasive and more adaptable manner.

Multiphoton intravital microscopy (MP-IVM) was performed between days 6 and 11 following the implantation of nephron sheets to evaluate their potential filtration function. Prior to imaging, we injected low- and high-molecular-weight dextran conjugated with FITC or Cy5 (LMW, 3 kDa-FITC / HMW, 500 kDa-Cy5) into the systemic blood circulation via intravenous injection (Fig. 6D and Supplementary Fig. 7B). To distinguish the implanted nephron tissues from the background, we generated a 3D-reconstructed surface of nephron structures by using the fluorescent dye Hoechst 33342 as a nuclear label, which was locally injected into the skinfold chamber, 5–10 min before administering the dextran (Fig. 6D, gray surface at the left image). Immediate imaging of the nephron sheets using multiphoton intravital microscopy suggested that the flow of HMW dextran was restricted to the 3D-reconstructed glomerular vasculature-like framework (Fig. 6D, center image), whereas LMW dextran dynamically travels into tubule-like structures and interstitium from the surrounding glomerular vasculature-like framework, embodying both HMW and LMW dextrans (Fig. 6D, right image, Supplementary Movie 1). Furthermore, the presence of LMW dextran accumulation in tubule-like cylindrical or interstitial structures within the organoids labeled with either Hoechst or wheat germ agglutinin (WGA)-AF647 conjugate suggested a potential filtration and/or secretion process through the organoid vasculature (Fig. 6D and Supplementary Fig. 8A–C; Supplementary Movie 1). Within 30–60 min post-dextran injection, we observed the pooling of LMW dextran, while HMW dextran did not show similar behavior, indicating the differential distribution of these molecules within the interstitial spaces of the nephron sheet (Fig. 6D and Supplementary Fig. 8A–C; Supplementary Movie 1).

Subsequently, the implanted tissues were surgically extracted for immunohistochemical assessment. Examination of extracted nephron sheets demonstrated their apparent invasion by the gross host vasculature network visible (Fig. 6E). Utilizing tissue clearing techniques following the fixation of the explanted sheets, their Z-stack images unveiled the transition from MECA-32+ (Mouse Pan-endothelial Cell Antigen-32 + ) mouse vessels to MECA32-CD146+ human endothelial networks (Fig. 6F and Supplementary Fig. 9A). Although the anti-CD146 monoclonal antibody can react with both human and murine endothelial cells (ECs), the MECA-32 monoclonal antibody is only specific to murine ECs. Thus, the lack of MECA-32 staining in particular CD146+ ECs suggested that they were human ECs, while the alignment of single positive MECA-32-CD146+ ECs (Fig. 6F, white arrows) with double positive MECA-32 + CD146+ ECs along these vessels represented the human-to-murine vessel anastomosis. This observation provided evidence of the successful anastomosis of human organoid vessels with the host’s circulatory system, facilitating the filtration of LMW dextran performed by implanted nephron sheets.

### The human glomerular capillary network in implanted nephron sheets provides the selective filtration and uptake of LMW dextran in proximal tubules

**Fig. 7:**
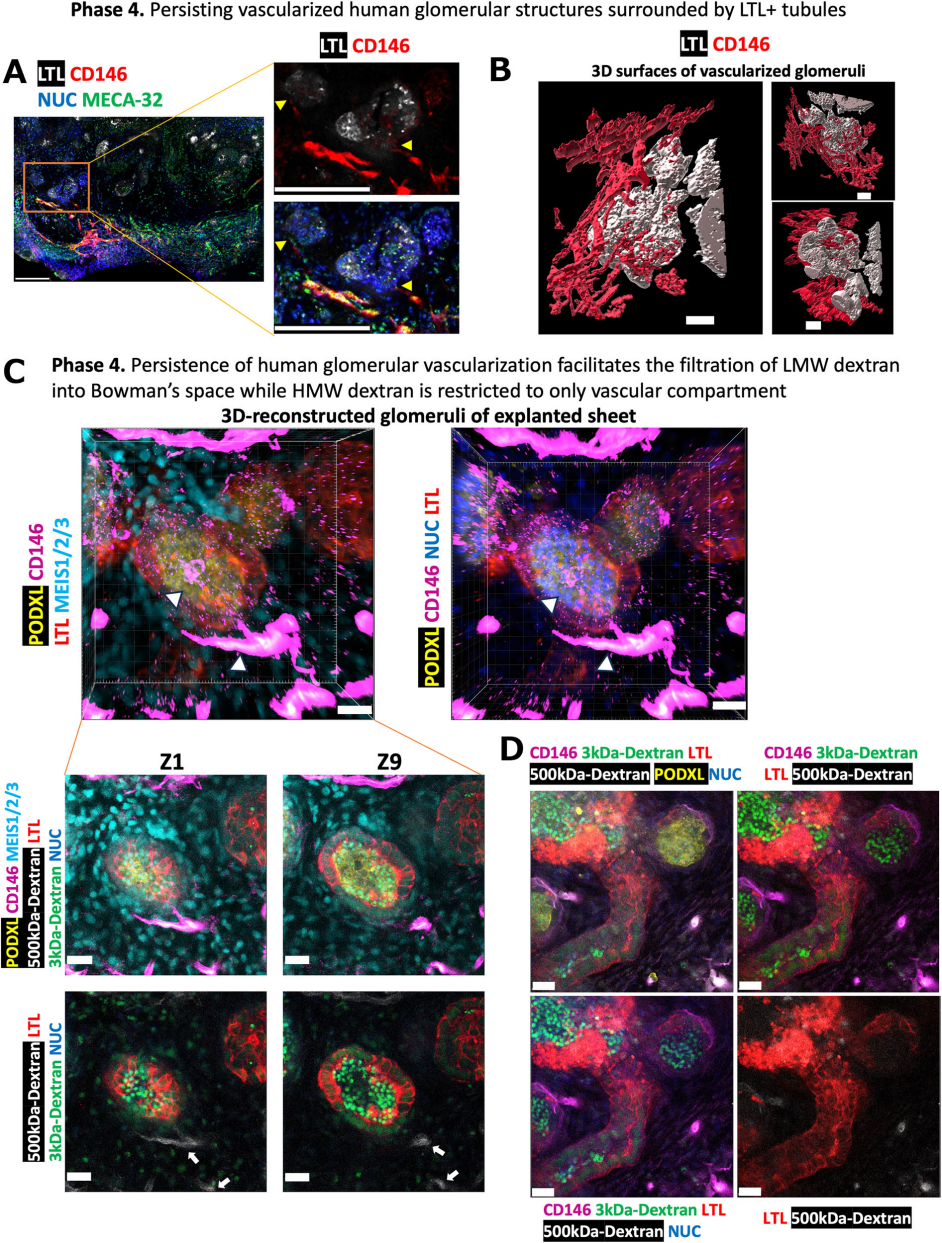
Implanted nephron sheets maintain human glomerular vascularization and exhibit selective filtration of dextrans. **A** Confocal microscopy images show the persistence of the CD146+ human endothelial network that is anastomosed with the MECA-32+ murine endothelial network. Yellow arrowheads indicate the human capillaries infiltrating the nephron structures. Scale bars: 150 μm. **B** 3D reconstructed surfaces showing human vessel penetration into nephron structures. Z-stack images were acquired at a step size of 5 μm. The scale bars: 50 μm. **C** 3D rendering of explanted nephron sheets indicates the persistence of the CD146+ human glomerular endothelial network supported by MEIS1/2/3+ stromal cells inside the podocyte clusters (markers: PODXL, CD146, LTL, MEIS1/2/3). The upper and lower white arrowheads pointed out the human glomerular capillary network and accompanying afferent arteriole, respectively. The Z-stack images of these nephron structures demonstrate the presence of LMW dextran in Bowman’s space and its uptake by proximal tubular epithelial cells and stromal cells, whereas HMW dextran (two white arrows) is detected only inside the vessels. Z-stack images were acquired with a step size of 2 μm, as Z1 represents the top stack of 3D structures. Scale bars: 20 μm. **D** Fixed tissue images showing a distinct distribution of LMW and HMW dextran, suggestive of filtration and flow of LMW dextran from vascular lumens to Bowman’s space and tubular lumens, in contrast to HMW dextran. Z-stack analysis revealed the uptake of LMW dextran by LTL+ proximal tubules and PODXL+ podocytes, while HMW dextran was strictly limited to CD146+ vascular lumen and absent from the tubules. Scale bars: 20 μm.

There are technical limitations of intravital live-cell imaging regarding the depth and the available number of co-staining parameters to distinguish the different compartments of the implanted nephrons. To complement the live-cell imaging in Fig. 6, we fixed the nephron sheets, followed by their tissue-clearing and immunostaining after their extraction from the murine dorsal skinfold chambers (DSFCs). Next, we generated 3D reconstructed images of these sheets by multiplex confocal microscopy to localize and distinguish low- and high-molecular-weight (LMW and HMW) dextrans inside glomerular, vascular, and tubular compartments of nephrons. These 3D images first revealed that the CD146+ human endothelial network of LTL+ Bowman’s capsule-like structures originated from peripheral CD146+ and MECA-32+ human-to-murine (graft-host) vessel anastomosis surrounding the nephron structures (Figs. 6F, 7A, B, and Supplementary Fig. 9A). Additionally, 3D-reconstructed surfaces and 2D frozen sections of these implanted nephron structures pointed out the persistence of CD146+ and EHD3+ human glomerular endothelial network surrounded by COL4A1+ glomerular basement membrane and polarized podocytes, and ITGA2+ stromal cells, underscoring the importance of pre-vascularization with human endothelia via mechanotransduction before implantation into animals (Fig. 7C, upper row, Supplementary Fig. 8D–G, Supplementary Movie 2). In accordance with the persisting human glomerular capillary network and its active filtration of LMW dextrans while sparing HMW ones during MP-IVM, further analysis of fixed and stained nephron sheets revealed the presence of LMW dextran in Bowman’s space, LTL+ proximal tubular epithelial cells, and surrounding stromal cells, whereas HMW dextran was strictly confined to vascular lumens including glomerular vascular structures, absent from the tubules (Fig. 7C, lower row and 7D, and Supplementary Fig. 9B and C).

To assess the potential diffusion of LMW dextran from tubular basal sides, we performed a live-cell imaging experiment of LMW dextran in vitro over one hour. While the rapid distribution of LMW dextran throughout the interstitial space was observed, tubular luminal and cytoplasmic signals were not increased (Supplementary Fig. 9D). Taken together, both live-cell imaging and fixed tissue analyses suggest that LMW dextran undergoes glomerular filtration and subsequent reabsorption into the interstitial space via tubules. The absence of HMW dextran in the interstitial space and tubules implies filtration selectivity retained within the organoid glomeruli.

## Discussion

In this study, we present a methodology for the large-scale production of vascularized kidney organoids that offers substantial reductions in cost and labor. This approach stands in stark contrast to existing protocols and obviates the need for culture vessel changes, enabling the uninterrupted use of a single bioreactor throughout the differentiation process^9,14,41^. This method leverages a compact stirred bioreactor, requiring a mere 5 ml of medium to generate a substantial quantity of nephron structures. To put this in perspective, the yield of nephron numbers from this small volume rivals that of multiple rodent kidneys or even a human kidney, underscoring the high efficiency of this process. A series of rigorous optimization experiments have revealed that the manipulation of spheroid size and the total organoid volume per bioreactor are pivotal parameters for ensuring successful differentiation within this 3D culture system. The resultant kidney organoids not only exhibit segmented nephron structures but also demonstrate well-developed interstitial stroma and robust vascular networks, although they were kept in the medium without any additional growth factors since day 13 of differentiation, based on our previous protocol^7,12^. This significant reduction in production costs, coupled with the significant increase in yield, holds promise for advancing research in disease studies and kidney regenerative medicine. The newfound scalability and cost-effectiveness of this method expand the horizons of human kidney organoid applications, ushering in a transformative era of scientific exploration in this critical domain.

Of particular interest, improved glomerular vascularization of podocyte clusters and tubular maturation of STR organoids further add significance to this study. Previous studies have demonstrated the effect of fluid flow on interstitial and glomerular vascular formation in organ-on-chip systems^26,42^. However, the production of large quantities of vascularized kidney organoids has not been feasible with the available methodologies. Additionally, the mechanisms by which glomerular vascularization is facilitated have been of great interest in devising strategies for the development of functional kidney tissues for urinary production. In this study, we observed a significant upregulation of both integrin expression and their downstream patterns associated with ECM and actin cytoskeletal network reorganization in both endothelial, nephron epithelial, and mesenchymal compartments of STR organoids when compared to static organoids, as detected by our bulk and scRNA-seq. Following these findings, we postulated that integrins, required for functional mechanotransduction machinery, may mediate the glomerular vascularization process via mechanotransduction induced by mechanical stress during the early stage of nephron formation^30,34^. Additionally, the large size of delta-wing-shaped impellers in 5 ml-STR wells, unlike traditional magnet stirrers, may also contribute to the mechanical forces applied to vascularizing STR organoids as they flow around the blades of the impellers. This is in contrast to previous studies, which used 125 ml-spinner flasks with much smaller impellers^14^. On the other hand, the degree of glomerular capillary loop formation, presumably driven by fluidic shear stress, is less than that observed in native kidneys. It is hypothesized that mechanical shear stress, particularly within the vascular lumen, is necessary to further enhance capillary loop formation to the extent found in native human kidneys.

Integrin α and β subunits are expressed in the form of obligate heterodimers on cell membranes to bind to extracellular matrix components such as collagen and fibronectin for cell survival, cytoskeletal regulation, and the preservation of cell polarity and tissue integrity^33^. Integrin α2 component is highly expressed by mesangial, distal, or connecting tubular cells^31^. A review of the murine studies pointed out the controversial reports about the kidney phenotypes (no defect versus accumulation of glomerular and tubulointerstitial matrix with proteinuria) of integrin α2-deficient mice^43,44^. Meanwhile, another integrin component, β1 subunit, expressed by mesangial and glomerular capillary endothelial cells^31^, has been shown to be critical for the development of the glomerular capillaries and the maintenance of glomerular structural integrity^35,36^. The genetic deletion of a specific protein complex, such as the integrin α2β1, via gene editing is not straightforward. As the integrin β1 subunit makes heterodimers with 12 different types of α subunits according to a recent report^29^, the possible ablation of integrin β1 could affect the signaling of 12 different integrin heterodimers, disrupting the whole differentiation process. Thus, to interrogate the function of mesangial α2β1 integrin, we followed a chemical approach by the application of BTT3033, a sulfonamide derivative, which exerts its inhibitory effect on the downstream signaling cascade of integrin α2β1 complex but not β1 subunit alone^45,46^. In this context, these perturbation experiments demonstrate the crucial role of integrin signaling in the promotion of glomerular vascularization during the early stage of nephron formation, complementary to our bulk and scRNA-seq. Previous studies in the literature have so far detected that the integrin α2β1 complex can bind to Collagen types I-V and Laminin 1, 2, and 5 components of the extracellular matrix with different binding avidity^47–53^. Future studies should focus on the signaling cascade involving the integrin α2β1 complex of mesenchymal/mesangial cells and collagen/laminin components of the glomerular basement membrane in order to find alternative ways to enhance organoid glomerular vascularization without mechanical stimuli in vitro.

Leveraging the combination of enhanced glomerular vascularization and tubular maturation, we have determined that the fabrication and implantation of large nephron sheets in mice are feasible without tissue necrotic degradation. It is noteworthy that current approaches aimed at enhancing organoid vascularization through animal transplantation result in chimeric tissue formation with host endothelia, which raises concerns about the fidelity of filtration function^54^. Our results, however, demonstrate the preservation of vascularized glomerular structures with human endothelia in NSG mice. Furthermore, the approach of implanting these sheets into the back skin of mice using dorsal skin chambers enables repetitive intravital imaging to assess tissue viability, blood circulation, and filtration function. Although it remains to be determined what the exact physiological threshold is for dextran size selectivity, the current experiments simply followed a previously published approach^55^ using two different sizes of dextran in animal experiments, and as of now, human data is not available. Notably, we observed the presence of LMD but not HMD in proximal tubules and the interstitial space, confirming filtration selectivity within the human vascularized glomeruli. This method holds promise for functional assessments of glomerular filtration, particularly in the context of podocytopathies for therapeutic evaluation. Additionally, single-cell RNA sequencing and functional assays revealed marked improvements in tubular maturation and function in STR-cultured organoids, including enhanced tubular transport of organic cations. Meanwhile, our kidney organoids exhibit the differentiation status of metanephric mesenchyme originated from posterior intermediate mesoderm without the differentiation of ureteric buds responsible for the generation of collecting duct systems. Thus, these organoids lack the ability to drain urine due to the absence of collecting ducts, which would receive the urine flow from the connecting tubules of these kidney organoids.

Lastly, our scRNA-seq analysis was limited by the freezing and shipping process of single-cell suspensions, which hindered robust comparisons with existing datasets from fetal and adult human kidneys and kidney organoids, even when applying batch correction methods such as Harmony^56^. To comprehensively assess the maturation status of STR organoids relative to human kidney tissue, further studies are needed to address batch effects arising from variations in library preparation, sequencing depth, normalization methods, reference genome annotations, and analysis pipelines, and to evaluate in-depth batch variations and degenerative changes during long-term culture with a large cohort of multiple batches using comprehensive assays such as comparative scRNA-seq analysis of multiple batches.

In summary, we have developed an optimized method for kidney organoid production that enables the generation of substantial quantities of vascularized nephron structures. This innovative approach permits the creation of implantable nephron sheets, presenting a novel model for the assessment of filtration function within human vascularized glomeruli. The outcomes of this research hold the potential to contribute significantly to the advancement of implantable bioengineered kidneys at GMP grade for kidney replacement therapy in the future.

## Methods

*KEY RESOURCES TABLE*

**Table:** 

REAGENT or RESOURCE	SOURCE	IDENTIFIER
**Antibodies**		
Anti-Actin, α-Smooth Muscle - Cy3 conjugated antibody	Sigma-Aldrich	C6198, RRID: AB_476856
Beta III TUBULIN	Millipore	MAB1637, RRID: AB_2210524
CD146	Abcam	ab75769, RRID: AB_2143375
CD31	Abcam	ab9498, RRID: AB_307284
CDH1	Abcam	ab11512, RRID: AB_298118
Collagen IV α5 (B51 and H53 clones)	Shigei Medical Research Institute	SGE-C451 (B51), RRID: AB_2924380 and SGE-C453 (H53)
Collagen IV α1	Rockland	600-401-106S, RRID: AB_11183330
EHD3	Novus Biologicals	31896
Integrin alpha2 (EPR5788)	GeneTex	GTX63576
LTL	Vector lab	B-1325, RRID: AB_2336558
LTL-Alexa Fluor 647	Bioworld	21511594
MECA-32	BD Biosciences	553849
MEIS1	Active motif	39796
MEIS1/2/3	Santa cruz	sc-101850, RRID: AB_2143143
NEPHRIN (NPHS1)	PROGEN	GP-N2, RRID: AB_2904121
PAX2	Biolegend	901001, RRID: AB_2565001
PDGFR-β	R&D systems	AF385, RRID: AB_355339
NPHS2	Abcam	ab50339, RRID: AB_882097
PODXL	R&D systems	AF1658, RRID: AB_354920
SALL1	R&D systems	PP-K9814-00, RRID: AB_1964373
SIX2	Proteintech	11562-1-AP, RRID: AB_2189084
WT1	Santa cruz	sc-192, RRID: AB_632611
**Chemicals, peptides, recombinant proteins, consumables**		
StemFit® Basic02	Amsbio	SFB-500
StemFit® Basic04 complete	Amsbio	SFB-504-CT
LDEV-Free hESC-qualified Geltrex	Life Technologies	A1413302
AccutaseTM	STEMCELL TECHNOLOGIES	07920
PBS	Life Technologies	10010-023
24-well tissue culture plates	TPP	92024
Y27632	TOCRIS	1254
FGF2	Peprotech	100-18B
aRPMI1640	Life Technologies	12633-020
L-GlutaMAX	Life Technologies	35050-061
CHIR99021	TOCRIS	4423
Dorsomorphin	TOCRIS	3093
Activin	R&D	338-AC-050
96-well, round-bottom, ultra-low attachment plates	Corning	7007
5 ml bioreactor	Reprocell	ABBWVS05A
FGF9	R&D	273-F9-025/CF
30 ml bioreactor	Reprocell	ABBWVS03A
μ-slide 8-well glass-bottom chamber slides	Ibidi	80827
Rhodamine 123	Sigma	83702
3 kDa dextran conjugated with Fluorescein	Thermo Fisher Scientific	D3306
BTT3033	TOCRIS	4724
0.25% Trypsin-EDTA	Thermo Fisher Scientific	25200056
70-μm cell strainer	Greiner Bio-One	542070
500 kDa Dextran conjugated with Cyanine 5	Nanocs	DX500-S5-1
TRIzol	Thermo Fisher Scientific	15596018
High-capacity cDNA Reverse Transcription kit	Thermo Fisher Scientific	4368813
iTaq SYBR green supermix	Bio-Rad	172-5121
Streptavidin/biotin blocking kit	Vector Laboratories	SP-2002
VECTASHIELD antifade mounting medium	Vector Laboratories	H-1000, H-1200-10
IHC Antigen Retrieval Solution	Thermo Fisher Scientific	00-4955-58
99% extra-pure p-Xylene	Thermo Fisher Scientific	220210025
**Cell lines**		
WA09(H9) human ESCs	WiCell	WAe009-A, RRID: CVCL_9773
BJFF.6 human iPSCs	Sanjay Jain, WUSTL	BJFF.6, RRID: CVCL_VU02
HUES62 human ESCs	Harvard Stem Cell Institute iPS Core (Bastepe Lab)	RRID: CVCL_B197
**Deposited data**		
Bulk RNA-seq	DDBJ Sequence Read Archive	DRA010266
Single-cell RNA-seq	GEO	GSE271428

### Maintenance of hPSCs

H9 human ESCs (passage 30–50) and BJFF. 6 human iPSCs (passage 30–50) were maintained in either StemFit® Basic02 (Ajinomoto, Japan) supplemented with FGF2 (10 ng/ml) (Peprotech, #100-18B) or StemFit® Basic04 Complete type (Ajinomoto, Japan) in 6-well tissue culture plates (Falcon, #353046) coated with 1% vol/ LDEV-Free hESC-qualified Geltrex (Life Technologies, #A1413302) in a 37°C incubator with 5% CO2. These hPSCs were passaged using Accutase^TM^ (STEMCELL TECHNOLOGIES^TM^, #07920) and seeded again at a density of 4-18 × 10^3^ cells/well every 7 days according to the manufacturer’s protocol. The H9 cell line was purchased from WiCell, and the BJFF. 6 cell line was kindly provided by Dr. Sanjay Jain at Washington University. The HUES62 cell line is an established human embryonic stem cell line found in the NIH Human Embryonic Stem Cell Registry (registration number: 0065; approval number: NIHhESC-10-0065), which was authenticated by the Harvard Stem Cell Institute iPS Core (https://ipscore.hsci.harvard.edu/) and kindly provided by Dr. Murat Bastepe from Massachusetts General Hospital.

### Differentiation of hPSCs in static culture for 3D kidney organoid formation

hPSCs grown on Geltrex were washed once with PBS (Life Technologies, #10010-023) and dissociated into single cells with Accutase. Cells were then plated at a density of 0.8-1.4 × 10^4^ cells/well onto 24-well tissue culture plates (TPP, #92024) coated with 1% Geltrex in StemFit® Basic02 or Complet04 supplemented with the ROCK inhibitor Y27632 (10 µM) (TOCRIS, #1254) and FGF2 (10 ng/ml). After 72 hours, cells (approximately 50% confluent) were briefly washed in PBS and then cultured in the basic differentiation medium consisting of advanced RPMI (aRPMI) 1640 (Life Technologies, #12633-020) and 1X L-GlutaMAX (Life Technologies, #35050-061), supplemented with CHIR99021 (CHIR) at a final concentration of 5-*8* µM (TOCRIS, #4423) with or without Dorsomorphin at a final concentration of 100 nM (TOCRIS, 3093) for 4 days to induce late primitive streak cells. To induce posterior intermediate mesoderm, cells were then cultured in the basic differentiation medium supplemented with activin at a final concentration of 10 ng/ml (R&D, #338-AC-050 or Ajinomoto, Tokyo) for 2 to 3 days. For the induction of nephron progenitor cells, their medium was then refreshed with the addition of FGF9 at a final concentration of 10 ng/ml (R&D, #273-F9–025/CF or Ajinomoto, Tokyo) for one day of incubation. On day 7 or 8 of differentiation, metanephric mesenchyme cells, differentiated from hPSCs, were dissociated with Accutase and resuspended in the basic differentiation medium supplemented with CHIR (2.5-3 µM) and FGF9 (10 ng/ml). Next, these cells were placed in 96-well, round-bottom, ultra-low attachment plates (Corning, #7007) at a seeding density of 1 × 10^5^ cells per well. The plates were centrifuged at 240 rpm for 60-90 seconds and then incubated in a 37°C incubator with 5% CO2 for 2 days. The medium was changed to the basic differentiation medium supplemented with FGF9 10 ng/ml and cultured for 4 more days. Finally, day 14 organoids were cultured in a basic differentiation medium with no additional factors until they became day 21–63.

### Differentiation of H9 human ESCs into kidney organoids in the STR system

For the optimization of the differentiation protocol, cells were first seeded at a density of 2, 4, and 8 × 10^5^ (H9) cells/ml into each of 5 ml vessels (Reprocell, #ABBWVS05A) in StemFit® Basic02 supplemented with the ROCK inhibitor Y27632 (10 µM) and FGF2 (10 ng/ml). Next, the seeding density of 2 × 10^5^ cells/ml was applied for the following STR differentiation of H9 kidney organoids. The medium was replaced every day. After 96 hours, cells were briefly washed in PBS and then cultured in the basic differentiation medium (aRPMI 1640 including 1X L-GlutaMAX) supplemented with CHIR99021 (3, 5, 7, or 9 µM) for 4 days. Cells were then cultured in the basic differentiation medium supplemented with activin (10 ng/ml) for 3 days. For the induction of nephron progenitor cells, the media were then changed to the basic differentiation medium supplemented with FGF9 (10 ng/ml) for a day. NPCs were further differentiated with CHIR (3 µM) and FGF9 (10 ng/ml) for 2 days. The medium was then changed to the basic differentiation medium supplemented with FGF9 10 ng/ml and cultured for 4 more days. After that, the organoids were cultured in a basic differentiation medium with no additional factors until they reached day 21–49. The medium was replaced every 2 or 3 days. During the medium and supplementation changes, 5-10% of organoids were removed if the medium color of the particular vessel was yellow due to high confluency of the organoids.

### Differentiation of BJFF.6 human iPSCs into kidney organoids in the STR system

Cells were seeded at a density of 1–3 × 10^5^ cells/ml into disposable 5 ml STR wells (Reprocell, #ABBWVS05A) or 2 × 10^5^ cells/ml into disposable 30 ml bioreactor (Reprocell, ABBWVS03A) with a stirring rate of 80 rpm in the presence of StemFit® Basic04 Complete type medium supplemented with the ROCK inhibitor Y27632 at a final concentration of 10 µM. After 24 h, each STR well was split into 2 wells with the addition of 2.5 ml medium to each (half medium change). After the next 24 h, spheroids were briefly washed in PBS by centrifugation at 100 × *g* for 3 min, and then gently resuspended and cultured in the basic differentiation medium (aRPMI 1640 and 1X L-GlutaMAX) supplemented with CHIR99021 (4.5, 5.5, and 6.5 µM) and Dorsomorphin (100 nM, TOCRIS, 3093) for 4 days. After day 0 of differentiation, we refreshed the differentiation medium of STR spheroids/organoids without the need for centrifugation. Cells were then cultured in the basic differentiation medium supplemented with activin (10 ng/ml) for 2 days. For the induction of nephron progenitor cells, the media were then changed to the basic differentiation medium supplemented with FGF9 (10 ng/ml) for a day. NPCs were further differentiated with CHIR (2.5 µM) and FGF9 (10 ng/ml) for 2 more days. Later, their medium was refreshed in the presence of only FGF9 (10 ng/ml) and cultured for 4 days. On day 13, organoids were cultured in a basic differentiation medium with no additional factors until they became day 70. Until day 13, their medium was daily replaced, while it was replenished every 2 days with a stirring rate of 120 rpm starting on day 14. During the replenishment of medium and its supplementation, if the medium color of the organoids was yellow due to their high confluency, 5–10% of organoids were removed together with the old medium.

Based on our hands-on experience, the optimization of cell seeding density for each cell line is important for successful differentiation. The concentrations of growth factors and the necessity of dorsomorphin remain the same as in our previously published static protocol. Therefore, transitioning from the previous static method to the new STR method does not require re-optimizing growth factors. Notably, the agitation rate does not need optimization when using the same delta-wing bioreactors described in the methods section.

### Transepithelial Rhodamine 123 (Rh123) cation transport assay

Two independent batches of STR organoids derived from BJFF.6 and HUES62 cells were used for this particular assay. To use static controls of STR organoids from the same batch, STR organoids derived from BJFF.6 cells were generated and kept in bioreactors starting from day 0 of differentiation until day 38, while their same-batch static controls were transferred from STR wells into static 96-well plates on day 14. Meanwhile, STR organoids derived from HUES62 cells were transferred from 96-well plates into bioreactors on day 14, while their same-batch static controls were kept in 96-well plates until day 32. First, μ-slide 8-well glass-bottom chamber slides (Ibidi, 80827) were coated with 1% Geltrex at 37 °C for 1 h. Upon removal of the Geltrex coating solution in DMEM, day 32–38 STR and static-control organoids were transferred to the coated 8-well chamber slides in aRPMI with 1% L-GlutaMAX. Next, organoid proximal tubules in the chamber slides were labeled with LTL conjugated with Alexa Fluor 647 (Bioworld, 21511594) for time-lapse imaging at a dilution ratio of 1:100 at 37 °C overnight. On days 36–40 of differentiation, the chamber slides containing LTL-labeled STR and static organoids were placed into the stage-top incubation system (Tokai Hit, STXG-WSKMX) installed on the confocal microscope (Leica STELLARIS 8). At the initial phase of their incubation at 37 °C with 5% CO2, LTL-labeled proximal tubules were first assigned and mapped by the confocal microscope. After we captured the initial images, the cation Rhodamine 123 (Rh123, Sigma, 83702, 10 mM) was added to the culture media at a final concentration of 10 μM (1:1000 dilution). Real-time imaging was immediately started to check the efficiency of cation transport through the proximal tubular epithelial cells. We used the Leica Application Suite X (LAS X) software platform to quantify the apical and basal Rh123 fluorescence intensity of proximal tubules by the line profile (10 μm-width) and histogram analysis. The apicobasal ratio of Rh123 fluorescence intensity was calculated after 20–30 min, once proximal tubular epithelial cells reached saturation, to assess the efficiency of cation transport from the basal to the apical side.

### In vitro 3 kDa dextran uptake assay

STR organoids derived from BJFF.6 cells were used for this particular assay. First, μ-slide 8-well glass-bottom chamber slides (Ibidi, 80827) were coated with 1% Geltrex at 37 °C for 1 h. Upon removal of the Geltrex coating solution in DMEM, day 38 STR organoids were transferred to the coated 8-well chamber slides in aRPMI with 1% L- GlutaMAX. The next day, organoid proximal tubules in the chamber slides were labeled with Wheat Germ Agglutinin (WGA) conjugated with Alexa Fluor 555 (WGA-AF555, Thermo Fisher Scientific, W32464, final concentration of 5 μg/ml) and LTL-Alexa Fluor 647 (Bioworld, 21511594) at 37 °C overnight at dilution ratios of 1:200 and 1:100, respectively. On day 40 of differentiation, the chamber slides containing LTL-labeled STR organoids were placed into the stage-top incubation system (Tokai Hit, STXG-WSKMX) installed on the confocal microscope (Leica STELLARIS 8). At the initial phase of their incubation at 37 °C with 5% CO2, LTL-labeled proximal tubules were first assigned and mapped by the confocal microscope. After we captured the initial images, 3 kDa dextran conjugated with Fluorescein (Thermo Fisher Scientific, D3306, 10 mg/ml) was added into the organoid media at a final concentration of 10 μg/ml (1:1000 dilution). The time-lapse imaging was performed for 1 h after the addition of 3 kDa dextran.

### Extracellular Matrix (ECM) exposure to nephron organoids

Tested ECMs were diluted in a cold medium with concentrations as follows: Fibrinogen (Sigma) 30 ng/ml, Laminin iMatrix-511 (Nippi) 2.4 μg/ml, Gelatin 0.5% (Sigma), and Matrigel® Matrix for Organoid Culture (Corning) 2%. SIX2+ cells were harvested and suspended in a medium containing diluted ECMs with a seeding density of 100 K cells/well into 96-well ultra-low attachment plates (Corning). Medium was refreshed every other day with no additional ECM supplementation.

### Treatment of STR organoids with the α2β1 integrin inhibitor

Day 14 STR organoids were treated with an inhibitor of α2β1 integrin, BTT3033 (TOCRIS, 10 mg, #4724 with a stock concentration of 10 mM dissolved in DMSO) with a final concentration of 1 μM for 7 and 18 days, respectively. Meanwhile, DMSO was applied as the vehicle treatment to the control group at 1:10,000 dilution, the same as the final concentration of DMSO in BTT3033-treated organoids. Following the completion of treatment, organoids were collected and fixed with 4% paraformaldehyde (PFA) to perform the immunostaining of the target proteins (CD146, CD31, PODXL, LTL, CDH1, MEIS1/2/3).

### Single-cell RNA-sequencing

STR organoids differentiated from H9 cells were transferred from the static condition into bioreactors on day 14 of differentiation, and their static counterparts were collected for dissociation into single cells on the differentiation day 36. After these organoids were washed with d-PBS, we treated them with 0.25% Trypsin-EDTA (Thermo Fisher Scientific, 25200056) by sequentially mixing and incubating at 37 °C for a total duration of 14 min. Following trypsinization, dissociated organoid cells were washed with aRPMI medium containing 1X L-GlutaMAX and 5% heat-inactivated fetal bovine serum (Thermo Fisher Scientific, A3840101) for the inactivation of trypsin. Single cell suspensions were then prepared by filtration through 70-μm cell strainers (Greiner Bio-One, 542070). After they were again washed with aRPMI medium containing 1X L-GlutaMAX, these single cells were cryopreserved in aRPMI medium containing 1X L-GlutaMAX and 10% DMSO. During the two washing steps, cells were centrifuged at 200 × *g* for 4 min at room temperature. Cryopreserved samples were kept at −150 °C until they were sent to the laboratory of Novogene Corporation in dry ice. In the Novogene lab, RNA from single cells was encapsulated, barcoded, and reverse-transcribed on a 10x Chromium Single Cell Platform (3’ ver3, 10x Genomics). The library was sequenced using the NovaSeq X Plus Series (PE150). The Cell Ranger v3 pipeline was utilized for sample demultiplexing, alignment based on the GRCh38 human genome assembly, filtering, unique molecular identifier (UMI) counting, single-cell 3’ end gene counting, and quality control according to the manufacturer’s parameters. Seurat, aided by OmicsBox 3.2.9, was used to analyze and visualize the data^38^. Given concerns about potential cellular stress caused by freezing, we tested a filtering step using a freezing stress marker, ABRAXAS2 (GO: 0071497). While ABRAXAS2 was detected throughout the cell populations, the clustering results were almost identical with or without filtering out ABRAXAS2+ cells, which accounted for less than 20% of the total cells. Therefore, differentially expressed gene analyses were conducted after filtering out ABRAXAS2+ cells.

### Generation and implantation of nephron sheets into mice

Implanted nephron sheets were generated from day 24 STR organoids by simple embedment of organoid collections in wide-bore tips (a volume range of 25–100 μl) onto a transwell plate without any exogenous ECM. Hence, the formation of nephron sheets was facilitated via the self-fusion of these organoids solely based on the capacity of their endogenous ECMs. The nephron sheets were kept at 37 °C for 4 days. Their medium was refreshed with aRPMI 1640 once every two days. One day before the implantation, a dorsal skin fold chamber (DSFC) was surgically installed onto each of the immunodeficient NOD/SCID/IL-2 receptor common gamma chain-deficient (NSG) mice. These DSFCs provided convenient platforms for the implantation of nephron sheets into the murine subcutaneous tissue, providing easy access to later stages of multiphoton intravital microscopy (MP-IVM). On day 28, the nephron sheets were either directly fixed for characterization by immunostaining or directly implanted into DSFCs of host NSG mice after being removed from their transwell membranes. Between days 6 and 11 of implantation, the function of nephron sheets in DSFCs was assessed by MP-IVM. After the completion of MP-IVM, implanted nephron sheets were explanted from DSFCs of mice and fixed with 4% PFA for immunostaining for imaging by confocal microscopy. Fixed nephron sheets were either spared for the whole-mount immunostaining or further processed for the generation of frozen or paraffin section blocks.

### Animals

Immunodeficient NOD/SCID/IL-2 receptor common gamma chain-deficient (NSG) mice were purchased from Jackson Laboratories and maintained in specific pathogen-free facilities at the Massachusetts General Hospital (MGH). Mice were included in experiments at the age of 6–12 weeks. Animals were cared for in accordance with the National Research Council’s Guide for the Care and Use of Laboratory Animals at Massachusetts General Hospital (MGH) in an AAALAC-accredited facility. All animal procedures were conducted in accordance with protocols approved by the MGH Institutional Animal Care and Use Committee under protocol 2022N000183 (Morizane). In addition, this study followed the ARRIVE Guidelines^57^.

### Preparation and follow-up of mice for multiphoton intravital microscopy

Dorsal skin fold chambers (DSFCs) were surgically installed at the back of NSG mice under general anesthesia with isoflurane, as described^58,59^. For analgesia, a subcutaneous 5 mg/kg dose of carprofen was administered to mice immediately before surgery and also every 24 h thereafter for 3 consecutive days. Intra- and perioperative anesthesia was applied using isoflurane inhalation.

### Multiphoton Intravital Microscopy

After DSFC-bearing mice were anesthetized with isoflurane, their DSFCs were mounted on a custom-built stage. Following the local injection of either WGA conjugated with Alexa Fluor 647 (WGA-AF647, Thermo Fisher Scientific, W32466) or Hoechst 33342 (Thermo Fisher Scientific, 62249) into the nephron sheet inside DSFC, we systemically injected a combination of 3 kilodalton (kDa) Dextran conjugated with Fluorescein (FITC) (fixable LMW dextran, Thermo Fisher Scientific, D3306) and 500 kDa Dextran conjugated with Cyanine 5 (HMW dextran, Nanocs, DX500-S5-1) in 100 μL d-PBS into NSG mice 10 min before the acquisition of images. The imaging depth varied within the range of 30–200 μm below the DSFC cover glass. A DeepSee HP and an Insight 3X Ti:sapphire lasers (Newport/Spectra-Physics) were tuned to 770 and 820 or 840 nm, respectively, for balanced multiphoton fluorescence excitation of Hoechst, FITC, Rhodamine, and AF647. Stacks of 5–15 optical sections (1024 × 1024 or 512 × 512 pixels) with 5–7 μm z-spacing were acquired every 30 s to visualize imaging volumes. Emitted light signals were detected through 460/50 nm, 525/50 nm, 595/50 nm, and 660/40 nm band-pass filters with non-descanned detectors. Datasets were transformed in IMARIS 10.0 (Bitplane) to generate maximum intensity projections (MIPs) for export as MP4 movies. Individual images were processed and exported with IMARIS 10.0.

### Transmission Electron Microscopy (TEM)

Day 35 and 70 organoids were fixed in 4% glutaraldehyde/0.1 M sodium cacodylate buffer (pH 7.4) for 3 h at room temperature on a gentle rotator, then placed into fresh 4% glutaraldehyde in cacodylate buffer, which continued to infiltrate the organoids overnight at 4 °C. Next, specimens were rinsed several times in 0.1 M cacodylate buffer, infiltrated in 1% osmium tetroxide for 1 h, rinsed several times again in cacodylate buffer, and then dehydrated through a graded series of ethanols to 100%. Following dehydration in ethanol, organoids were exposed to a brief dehydration in 100% propylene oxide for 10 min. Samples were then transferred into a 1:1 mix of propylene oxide:Eponate resin (Ted Pella, Redding, CA) for overnight infiltration at room temperature (in a hood) on a gentle rotator. The next day, specimens were placed into fresh 100% Eponate with three switch-outs occurring over the course of several hours, which were then transferred into BEEM capsules (Electron Microscopy Sciences #70000-B, size 00; Hatfield, PA) with fresh 100% Eponate resin and allowed to polymerize in a 60 °C oven for an incubation of 24-48 hours. Ultra-thin (70 nm) sections, which were cut by a Leica EM UC7 ultramicrotome, were then collected onto formvar-coated grids. Later, these specimens were stained with 2% uranyl acetate and Reynold’s lead citrate, and examined in a JEOL JEM 1011 transmission electron microscope at 80 kV. Finally, images were collected using an AMT digital imaging system with proprietary image capture software (Advanced Microscopy Techniques, Danvers, MA).

### qRT-PCR

Kidney organoids were collected with a pipette. RNAs were isolated from kidney organoid samples using TRIzol^TM^ (Thermo Fischer Scientific, 15596018) according to the manufacturer’s protocol. A minimum of 5 organoids is used per sample. cDNAs were synthesized using a High-Capacity cDNA Reverse Transcription kit (Thermo Fischer Scientific, 4368813). Quantitative Real-time PCR was performed using iTaq SYBR green supermix (Bio-Rad, 172–5121) and a Bio-Rad iQ5 Multicolor Real-time PCR Detection System. Primer sequences were designed using FASTA sequences (PubMed) and verified using Primer3, and one of the primers from the pairs of primers is designed to include an exon-exon junction. Target genes were normalized based on the expression of beta-actin (ACTB). The mRNA expression was calculated using the 2-ΔΔCt method, expressed as an n-fold difference relative to the control group, and reported with standard error bars. A full primer list can be found in Supplementary Table 1.

### Immunostaining

Immunostaining for confocal microscopy was performed to assess the protein localization of interest. Before immunostaining, each sample was washed with PBS 1X and then fixed for 1 h using 4% buffered PFA. Fixed organoids were either spared for whole-mount immunostaining or further processed for the generation of frozen blocks. After the fixative was removed with at least three consecutive washes in 1 ml PBS, whole-mount samples were blocked overnight at 4 °C or for 2 h at room temperature using 1 weight (wt) % donkey serum in PBS with 0.125 wt% TritonX-100 or 5% donkey serum with 1% bovine serum albumin (BSA) and 1% TritonX-100 in PBS. Next, samples were incubated with primary antibodies overnight at 4 °C in a solution of 0.5 wt% BSA and 0.125 wt% Triton X-100 or 1% BSA, 5% donkey serum, and 1% Triton X-100 in PBS at the dilution ratios listed in Supplementary Table 2. We used a streptavidin/biotin blocking kit (Vector Laboratories, SP-2002) to increase the staining efficacy of the biotin-labeled anti-LTL antibody according to the manufacturer’s instructions. Samples were then washed with PBS three times and left in PBS overnight. Secondary antibodies and nuclear dye (SYTOX Blue, Invitrogen, S11348) were applied to the samples at a 1:500 dilution ratio overnight at 4 °C in a buffer of 0.5 wt% BSA and 0.125 wt% Triton X-100 or 1% BSA, 5% donkey serum, and 1% Triton X-100 in PBS. After removal of the secondary antibody staining mix with three consecutive PBS washes, the samples were incubated with AF647-labeled MEIS1/2/3 conjugated antibody at room temperature for two hours. Samples were again washed in PBS three times prior to the tissue clearing procedure. If the nuclear dye SYTOX Blue was not included during the secondary antibody staining, samples were counter-stained with DAPI. For whole-mount staining of organoids and nephron sheets, tissue clearing of the samples was performed following the application of secondary antibodies and the conjugated MEIS1/2/3 antibody. They were fixed again with 4% PFA for 30–60 min at 4 °C. Following the removal of fixative by several washes with PBS, samples were dehydrated through a graded series of ethanols (50% and 70%) to 100% with a duration of 12–24 h for each ethanol concentration.

For the generation of frozen sections, after fixed organoids were washed with 1 ml of PBS three times, they were placed in 0.5-1 ml of 30% sucrose in PBS at 4 °C overnight. In the following days, organoids were placed in Epredia Peel-A-Way disposable tissue-embedding molds (Fisher Scientific, 2219), and after their excess fluid was aspirated, they were embedded in optimal cutting temperature freezing medium (Tissue-Plus O.C.T. Compound, Fisher Scientific, 23730571) inside the molds. Then, frozen blocks were generated using liquid nitrogen and acetone for a slow freezing process. Frozen blocks were stored at −20 °C or −80 °C freezer. Several days later, these blocks were cut using a cryostat in 10-μm sections and stored at −20 °C or −80 °C freezer. For immunostaining, subsequent to the thawing of frozen sections at room temperature, they were serially washed in Coplin jars containing PBS three times for 5 min. They were then blocked for 1–2 h using a blocking buffer, which contained 5% donkey serum and 0.3% Triton X-100 in PBS. The slides were immediately incubated with primary antibodies at 4 °C overnight in antibody-dilution buffer (ADB), which included 1% BSA and 0.3% Triton X-100 in PBS at the dilution ratios listed in Supplementary Table 2. The next morning, after the samples were serially washed with PBS three times, they were stained with Alexa Fluor secondary antibodies (Life Technologies) and counterstained with 4′,6-Diamidino-2-phenylindole (DAPI, Sigma-Aldrich, D8417) in ADB for 2 hours at room temperature, followed by a series of washes with PBS in three Coplin jars. Finally, samples were mounted with VECTASHIELD antifade mounting medium (Vector Laboratories, H-1000) with or without DAPI (Vector Laboratories, H-1200-10), and sealed using a cover slip. When staining with LTL-biotin, a streptavidin/biotin blocking kit (Vector Laboratories, SP-2002) was used as per the manufacturer’s protocol. Prior to the staining of the frozen sections of organoids and nephron sheets with the anti-EHD3 antibody (Novus Biologicals, 31896), which was designed for immunohistochemistry application, these slides were incubated with prewarmed 1X IHC Antigen Retrieval Solution (Thermo Fisher Scientific, 00-4955-58) for 2 min at 90 °C, followed by cooling down at room temperature. Afterward, these slides were serially washed with distilled water and PBS three times before proceeding with the blocking step.

When the nephron sheets were embedded in paraffin after their fixation step, these paraffin sections were then subject to deparaffinization in 99% extra-pure p-Xylene (Thermo Fisher Scientific, 220210025) (2 washes, 10 min each), followed by removal of xylene in graded 100%–75%–50%–25% ethanol (each wash for 5 min) and removal of ethanol in distilled water (3 washes, 5 min each). Then, these deparaffinized slides were incubated with prewarmed 1X IHC Antigen Retrieval Solution (Thermo Fisher Scientific, 00-4955-58) for 20 min at 90 °C, followed by cooling down at room temperature for 30 min. Afterward, these slides were serially washed with PBS three times before proceeding with the blocking step.

Immunofluorescence imaging of both 2D sections and 3D whole-mount organoids was performed using a Leica STELLARIS 8 confocal microscope.

### RNA-seq Library preparation and sequencing

RNA integrity was assessed (RNA Nano 6000 Assay Kit, Bioanalyzer 2100, Agilent Technologies, CA), and 10 ng-1 μg RNA per sample was used to generate sequencing libraries (NEBNext Ultra RNA Library Prep Kit for Illumina (New England Biolabs, USA)) according to the manufacturer’s recommendations. The libraries are pooled and loaded onto an Illumina NovaSeq 6000 S4 flow cell and are run with the manufacturer’s recommended settings.

### RNA-seq data analysis

Downstream analysis was performed using a combination of programs, including Spliced Transcripts Alignment to a Reference (STAR), HTseq, Cufflink, and our wrapped scripts. Differential expressions were determined through DESeq2/edgeR. GO and KEGG enrichment were implemented by the ClusterProfiler.

### Reads mapping to the reference genome

Reference genome and gene model annotation files were directly downloaded from the genome website browser (NCBI/UCSC/Ensembl). Indexes of the reference genome were built using STAR, and paired-end clean reads were aligned to the reference genome using STAR (v2.5). STAR used the method of Maximal Mappable Prefix (MMP), which could generate a precise mapping result for junction reads.

### Quantification of gene expression level

HTSeq v0.6.1 was used to count the number of reads mapped to each gene. Then, the FPKM of each gene was calculated based on the length of the gene, and the read counts were mapped to the corresponding genes. FPKM, reads per kilobase of exon model per million mapped reads, considers the effect of sequencing depth and gene length for the read counts at the same time, as it is regarded as the most common method for estimating gene expression levels^60^.

### Clustering

To identify the correlation between differences, we clustered different samples using expression level FPKM to see the correlation using the hierarchical clustering distance method with the function of heatmap, SOM (Self-organization mapping), and k-means using the silhouette coefficient to adapt the optimal classification with default parameters in R.

### GO analysis

Gene Ontology (GO) analyses were performed and graphically visualized using OmicsBox 3.2.9 and ShinyGO (version 0.77 and 0.82) (https://bioinformatics.sdstate.edu/go/)^23^. Acquired GO enrichment analyses were also confirmed with the Gene Ontology Resource (https://geneontology.org/). The significance threshold for the enrichment was set at a false discovery rate ≤ 0.05.

### 3D image analysis via reconstruction of 3D surfaces

3D image analysis was performed using IMARIS 10.0 (Oxford Instruments). Original image data files were converted to IMS files using IMARIS file converter 10.0. A machine learning classification method powered by Labkit, which is a Fiji plugin, was used for organoid segmentation to generate surfaces of organoid markers. In principle, pixel classifications were performed by the same algorithm in the same experiments, and obvious background noise was removed manually. Podocyte and endothelial surfaces of Day 32-35 STR and static organoids from BJFF.6 and HUES62 cell lines were generated by 3D surface reconstruction for the quantification of vascularized glomerular surfaces. STR organoids derived from BJFF.6 cells were generated and kept in bioreactors starting from day 0 of differentiation, while STR organoids derived from HUES62 cells were transferred into bioreactors from 96-well plates on day 14. For 3D surface reconstruction, we first formed a 3D surface of podocyte clusters through the segmentation of PODXL+ cells by IMARIS. Next, we masked the CD146 expression outside podocyte clusters, thereby keeping only CD146+ cells and structures localized within the whole surface of podocyte clusters. Finally, we generated the 3D vascular surface of CD146+ endothelial cells pertaining only to these podocyte clusters. The percentage of these vascularized podocyte clusters reflects the overall glomerular vascularization within organoids. Additionally, we defined another quantification parameter as the volume percent ratio of preformed vascular surface to the whole podocyte surface, which reflects the 3D surface changes of glomerular vascularization upon any applied treatment.

### Statistics

Data in all bar charts and dot plots are expressed as mean ± SD or ±SEM. The statistical analysis was done in GraphPad Prism 7, 8, and 9, and statistical significance was attributed to values of *p* ≤ 0.05 as determined by a two-tailed t-test or one-way ANOVA with Tukey’s multiple comparisons test. Different significance levels (*p* values) are indicated in each figure with asterisks: *p ≤ 0.05, **p ≤ 0.01, ***p ≤ 0.001, ****p ≤ 0.0001. For transparency, the raw data and sample numbers for all statistical analyses are provided as a supplementary raw data file (Supplementary Data 3).

### Ethics statement

This is a nonclinical laboratory study, which does not involve any patients and human participants or multi-region collaborations. In addition, this study does not possess any health, safety, security, or other risks to the public or participating researchers.

## Supplementary information

STR Supplement_Npj_251205 Clean FINAL

Supplementary Data1

Supplementary Data2

Supplementary Data3

Supplementary Movie1A

Supplementary Movie1B

Supplementary Movie2A

Supplementary Movie2B

## Data Availability

Bulk RNA sequencing data from this study have been submitted to the DDBJ Sequence Read Archive (DRA) under accession number DRA010266. In addition, single-cell RNA sequencing data have been deposited at GEO: GSE271428.

**Table:** 

Sample name	Experiment	Sample	Run
Day_0-1	DRX221400	DRS140453	DRR231145
Day_0-2	DRX221401	DRS140454	DRR231146
Day_0-3	DRX221402	DRS140455	DRR231147
NPC-1	DRX221403	DRS140456	DRR231148
NPC-2	DRX221404	DRS140457	DRR231149
NPC-3	DRX221405	DRS140458	DRR231150
Org_D21-1	DRX221406	DRS140459	DRR231151
Org_D21-2	DRX221407	DRS140460	DRR231152
Org_D21-3	DRX221408	DRS140461	DRR231153
Org_D35-1	DRX221409	DRS140462	DRR231154
Org_D35-2	DRX221410	DRS140463	DRR231155
Org_D35-3	DRX221411	DRS140464	DRR231156
Org_D49-1	DRX221412	DRS140465	DRR231157
Org_D49-2	DRX221413	DRS140466	DRR231158
Org_D49-3	DRX221414	DRS140467	DRR231159
Org_D63-1	DRX221415	DRS140468	DRR231160
Org_D63-2	DRX221416	DRS140469	DRR231161
Org_D63-3	DRX221417	DRS140470	DRR231162
STR_D21-1	DRX221418	DRS140471	DRR231163
STR_D21-2	DRX221419	DRS140472	DRR231164
STR_D21-3	DRX221420	DRS140473	DRR231165
STR_D35-1	DRX221421	DRS140474	DRR231166
STR_D35-2	DRX221422	DRS140475	DRR231167
STR_D35-3	DRX221423	DRS140476	DRR231168
STR_D49-1	DRX221424	DRS140477	DRR231169
STR_D49-2	DRX221425	DRS140478	DRR231170
STR_D49-3	DRX221426	DRS140479	DRR231171
