# Supplementary material for: Engineering scalable vascularized kidney organoids for in vivo glomerular filtration with human endothelial integration
Source: NPJ Biomed Innov. 2026 Feb 2;3:10. doi: 10.1038/s44385-025-00063-5 (PMC12864042; doi:10.1038/s44385-025-00063-5)
Supplement: Supplementary file 1 — STR Supplement_Npj_251205 Clean FINAL [file 44385_2025_63_MOESM1_ESM.pdf]

SUPPLEMENTAL MATERIAL

Supplementary Figure 1. Optimization of differentiation conditions: various seeding densities.

Supplementary Figure 2. Optimization of differentiation conditions into NPCs and kidney organoids: various CHIR concentrations in H9.

Supplementary Figure 3. Optimization of differentiation conditions in BJFF.6 in comparison to static organoids.

Supplementary Figure 4. Manufacturing cost and heatmap analysis of STR and static kidney organoid protocols.

Supplementary Figure 5. The ultrastructural architecture and vascularization pattern of ITGA2+ EHD3+ STR glomeruli with their COL4A1+ basement membrane.

Supplementary Figure 6. UMAP and GO term analysis of STR versus static organoids by single-cell RNA-sequencing.

Supplementary Figure 7. Effects of ECMs on nephron differentiation and the gating strategy of structures in intravital imaging.

Supplementary Figure 8. Intravital imaging of implanted nephron sheets and post-fixed tissue.

Supplementary Figure 9. Post-fixed images in implanted nephron sheets and live-cell imaging of STR organoids *in vitro*.

Supplementary Table 1. Primers for qPCR.

Supplementary Table 2. Primary antibodies.

Supplementary Table 3. The comparison of static and STR organoid protocols in terms of yield and materials in one plate (static) and one reactor (STR).

Supplementary Data 1 (separate Excel file). Differentially expressed genes and their GO term and ligand assessment in Endothelia 3 cluster (characteristics of glomerular endothelia).

Supplementary Data 2 (separate Excel file). Differentially expressed genes and their GO term analysis in STR Seurat clusters compared to static ones, including the gene lists in the heatmaps.

Supplementary Data 3 (Excel/PDF file). Raw data referring to the graphs and charts of the main and supplementary figures.

Supplementary Movie 1 (two MP4 files as 1A and 1B). Selective filtration of 3kDa dextran compared to 500kDa dextran.

Supplementary Movie 2 (two MP4 files as 2A and 2B). Human glomerular vascularization inside the explanted nephron sheet from murine DSFC.

Supplementary Table 1. Primers for qPCR.

Gene	Forward	Reverse
**ACTB**	CTCTTCCAGCCTTCCTTCCT	AGCACTGTGTTGGCGTACAG
**CD31**	TCATTACGGTCACAATGACGA	GAGTATCTGCTTTCCACGGC
**DMD**	GTGTGTCAACCTGTCTATCAAGG	CATGGCATCGTAGAAGTGGAAG
**MAP2**	CTCAGCACCGCTAACAGAGG	CATTGGCGCTTCGGACAAG
**MYOD1**	CGGCATGATGGACTACAGCG	CAGGCAGTCTAGGCTCGAC
**NEUROD1**	ATGACCAAATCGTACAGCGAG	GTTCATGGCTTCGAGGTCGT
**OAT1**	CTGGTTCTTCATTGAGTCGGC	GCCCGGAGTACCTCCATAC
**OCT2**	GATGGTTTTCGGCGTGCTT	TCGATGGTCTCAGGCAAAGC
**PAX2**	ACTCCATCAATGGGATCCTG	CCACACCACTCTGGGAATCT
**SALL1**	ACTACCGACCGAGACATCATTA	GGGAGGCCAACCACATAGTA
**SGLT2**	TGACCTACATGATTCCCTTGG	GACCAGGGAAATGCCAACTAT
**SIX2**	GCCTGCTCGACCCTACAGA	GCTTGTCAACTGCGGTTGC
**SLC3A1**	TTTTGCAGAAACACAATGAAGTC	CCAAGGAGAGGCGTGATAC
**WT1**	CTCCCCAAGACAGTGTAGGC	TACCAGGTTAAACTCGGTGACG

Supplementary Table 2. Primary antibodies.

Antibody	Source	Catalog or clone number	Dilution factor
**Anti-Actin, α-Smooth Muscle - Cy3 conjugated antibody**	Sigma-Aldrich	C6198	1:200
**Beta III TUBULIN**	Millipore	MAB1637	1:1000
**CD146**	Abcam	ab75769	1:200
**CD31**	Abcam	ab9498	1:200
**CDH1**	Abcam	ab11512	1:500
**Collagen IV α5 (B51 and H53 clones)**	Shigei Medical Research Institute	SGE-C451 (B51) and SGE-C453 (H53)	1:100
**Collagen IV a1**	Rockland	600-401-106S	1:200
**EHD3**	Novus Biologicals	31896	1:200
**Integrin alpha2 (EPR5788)**	GeneTex	GTX63576	1:200
**LTL**	Vector lab	B-1325	1:200
**LTL-Alexa Fluor 647**	Bioworld	21511594	1:100
**MECA-32**	BD Biosciences	553849	1:200
**MEIS1**	Active motif	39796	1:100
**MEIS1/2/3**	Santa cruz	sc-101850	1:200
**NEPHRIN (NPHS1)**	PROGEN	GP-N2	1:100
**PAX2**	Biolegend	901001	1:500
**PDGFR-β**	R&D systems	AF385	1:200
**NPHS2**	Abcam	ab50339	1:1000
**PODXL**	R&D systems	AF1658	1:500
**SALL1**	R&D systems	PP-K9814-00	1:100
**SIX2**	Proteintech	11562-1-AP	1:500
**WT1**	Santa cruz	sc-192	1:50

Supplementary Table 3. The comparison of static and STR organoid protocols in terms of yield and materials in one plate (static) and one reactor (STR).

(static: ml/plate, STR: ml/well)	Static	STR(5ml)	STR(30ml)
Amount of medium	19.2	5	30
Estimate organoids Volume (ul)	20	75	500
Estimate number of organoids	96	1150	10000
Estimate cost / 100 organoids($)	32.8	4.6	1.8

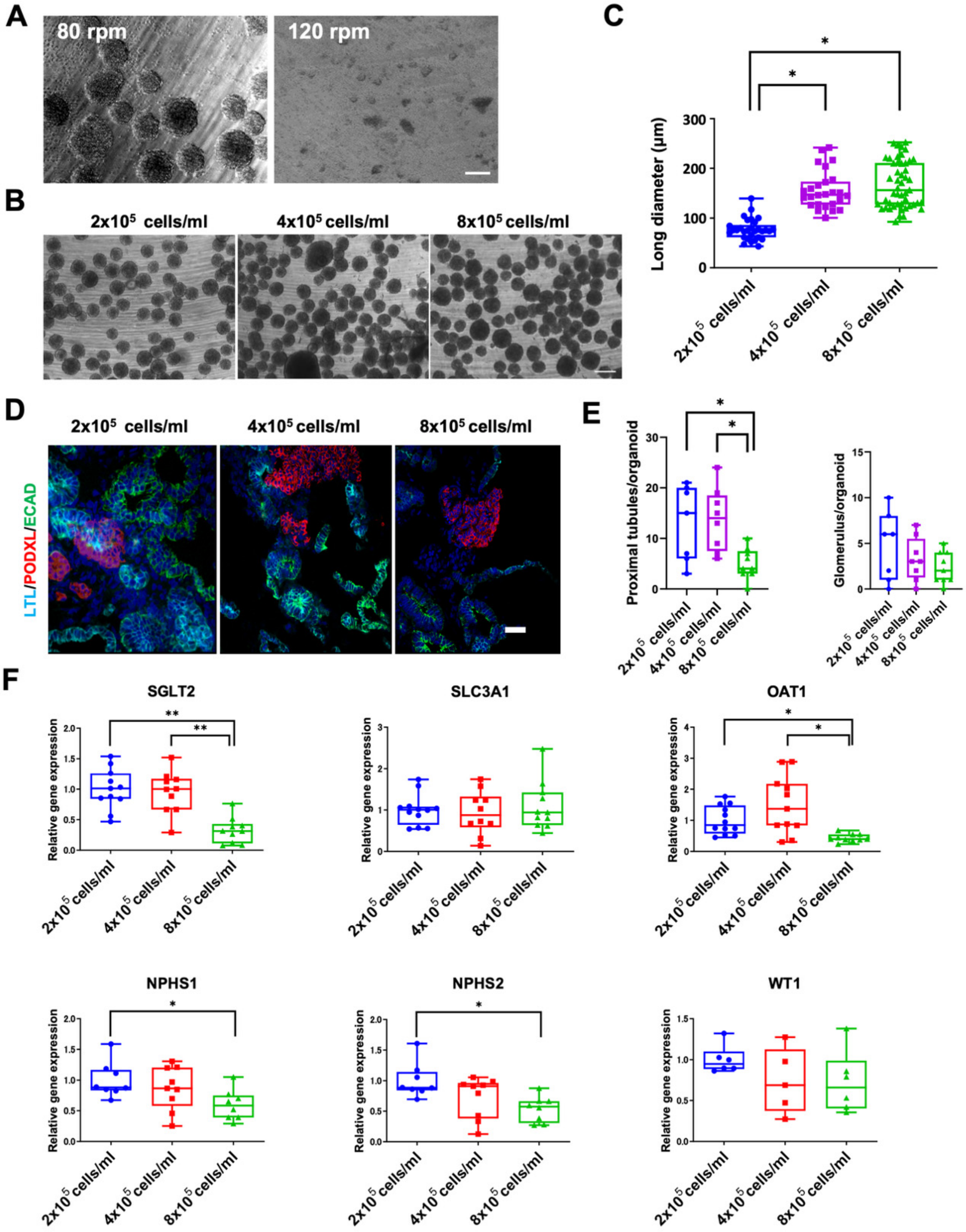
A
80 rpm
120 rpm
B
2x10^5 cells/ml
4x10^5 cells/ml
8x10^5 cells/ml
C
Long diameter (µm)
D
LTL/PODXL/ECAD
E
Proximal tubules/organoid
Glomerulus/organoid
F
SGLT2
SLC3A1
OAT1
NPHS1
NPHS2
WT1
Relative gene expression

Supplementary Figure 1. Optimization of differentiation conditions: various seeding densities (A) Brightfield images of day 8 aggregates generated with stirring rates of 80 and 120 rpm in STRs. Slower rates than 80 rpm failed to form spheroids. The scale bar represents 200 μm. (B) Brightfield images of hPSC aggregates on the differentiation day 0, which were generated at different seeding densities (2x, 4x, and 8x10^5 cells/ml). The scale bar represents 200 μm. (C) A box plot showing the size distribution of PSC aggregates on day 0 of differentiation. The long diameter was measured. n=26-34 spheroids in each condition. (D) Confocal images of day 21 organoids generated with 2x, 4x, and 8x10^5 cells/ml seeding densities. Podocytes (PODXL), proximal tubules (LTL), and distal tubules (ECAD). The scale bar represents 40 μm. (E) Box plots depicting the number of proximal tubules and glomerular structures in kidney organoids generated with various seeding cell densities. The structural numbers were manually counted per section of kidney organoids. n=7-9 organoids in each condition. (F) RT-qPCR for proximal tubule and podocyte genes in kidney organoids generated with varied seeding cell densities on day 21. n=6-12 organoids in each condition. Asterisks in the bar graphs (C, E, and F) indicate p values derived from two-tailed unpaired t-tests. Means ± S.D. *$p \leq 0.05$, **$p \leq 0.01$.

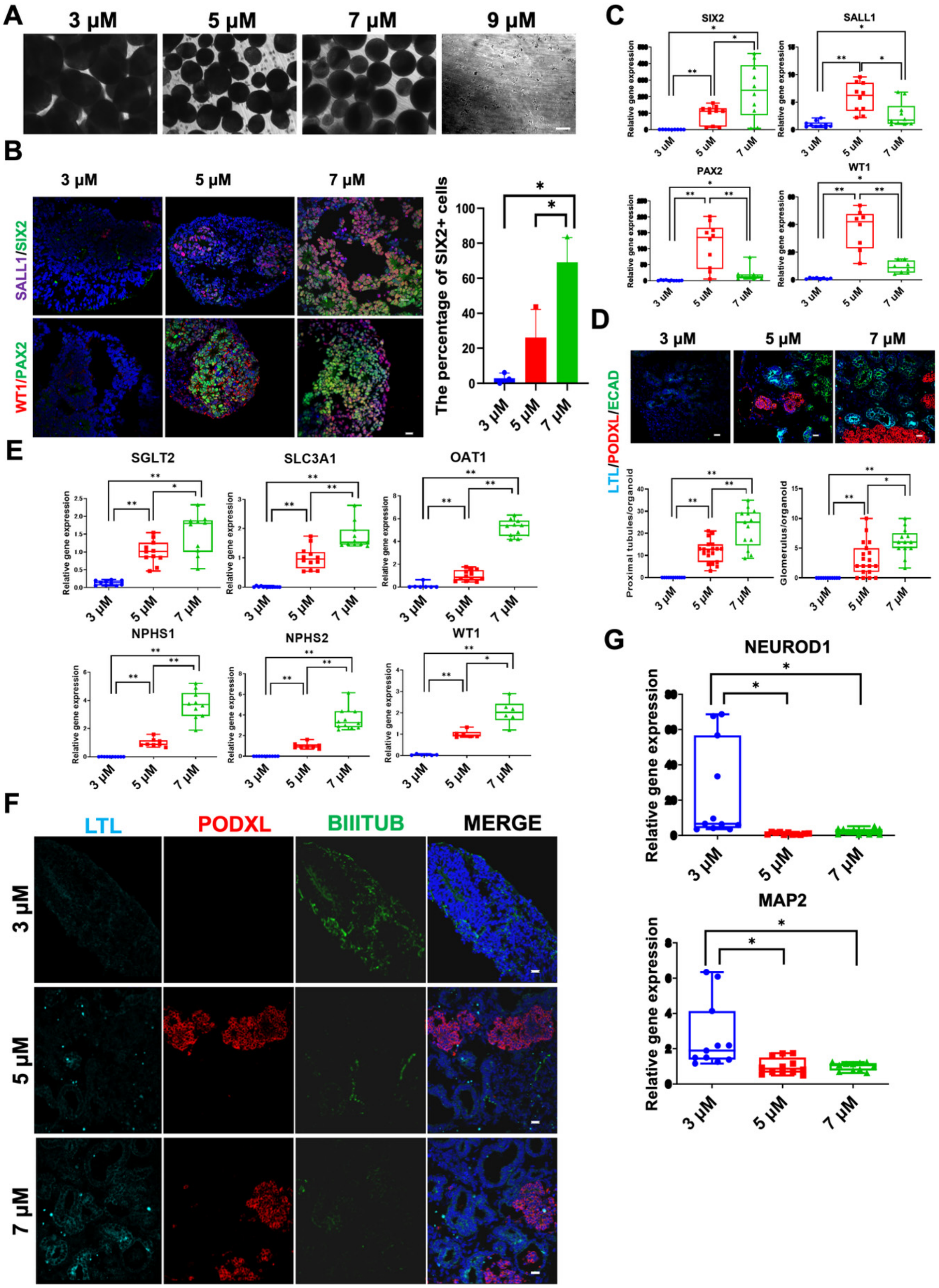
A
3 μM
5 μM
7 μM
9 μM
B
3 μM
5 μM
7 μM
SALL1/SIX2
WT1/PAX2
The percentage of SIX2+ cells
C
SIX2
SALL1
PAX2
WT1
Relative gene expression
D
3 μM
5 μM
7 μM
LTL/PODXL/ECAD
Proximal tubules/organoid
Glomerulus/organoid
E
SGLT2
SLC3A1
OAT1
NPHS1
NPHS2
WT1
Relative gene expression
F
LTL
PODXL
BIIITUB
MERGE
3 μM
5 μM
7 μM
G
NEURODI
MAP2
Relative gene expression

Supplementary Figure 2. Optimization of differentiation conditions for NPCs and kidney organoids: various CHIR concentrations in H9.

(A) Brightfield images of day 21 STR kidney organoids generated at the concentrations of 3, 5, 7, or 9 µM CHIR. The scale bar represents 200 µm. (B) Confocal images for nephron progenitor markers in the STR spheroids, consisting of nephron progenitor cells (NPCs) on day 8 of differentiation. The scale bars represent 20 µm. A bar graph showing the percentage of the SIX2+ cells in NPC spheroids on day 8. n=3 organoids. (C) The expression of metanephric mesenchyme genes by qRT-PCR in day 8 NPC aggregates at various CHIR concentrations. n=8-10 mRNA samples. (D) Confocal images of cryosectioned day 21 organoids at the concentrations of 3, 5, or 7 µM CHIR. The scale bar represents 20 µm. Box plots depict the number of proximal tubules and glomerulus-like structures in STR kidney organoids generated at the concentrations of 3, 5, or 7 µM CHIR. n=11-16 organoids. (E) The expression of tubular and podocyte genes by qRT-PCR in day 21 STR organoids generated within the same range (3-5-7 µM) of CHIR concentrations. n = 6-12 mRNA samples. Each mRNA sample was collected from 3-12 organoids. (F) Confocal images of cryosectioned day 21 STR organoids differentiated within the same range of CHIR concentrations. Podocytes (PODXL), proximal tubules (LTL), and neurons (BIIITUB: beta-III-tubulin). The scale bars represent 20 µm. (G) The expression of neuronal genes by qRT-PCR in day 21 STR organoids. n=11-12 mRNA samples. Each mRNA sample was collected from 3-12 organoids. Asterisks in the bar graphs (B, C, D, E, and G) indicate p values derived from two-tailed unpaired t-tests. Means ± S.D. *p ≤ 0.05, **p ≤ 0.01.

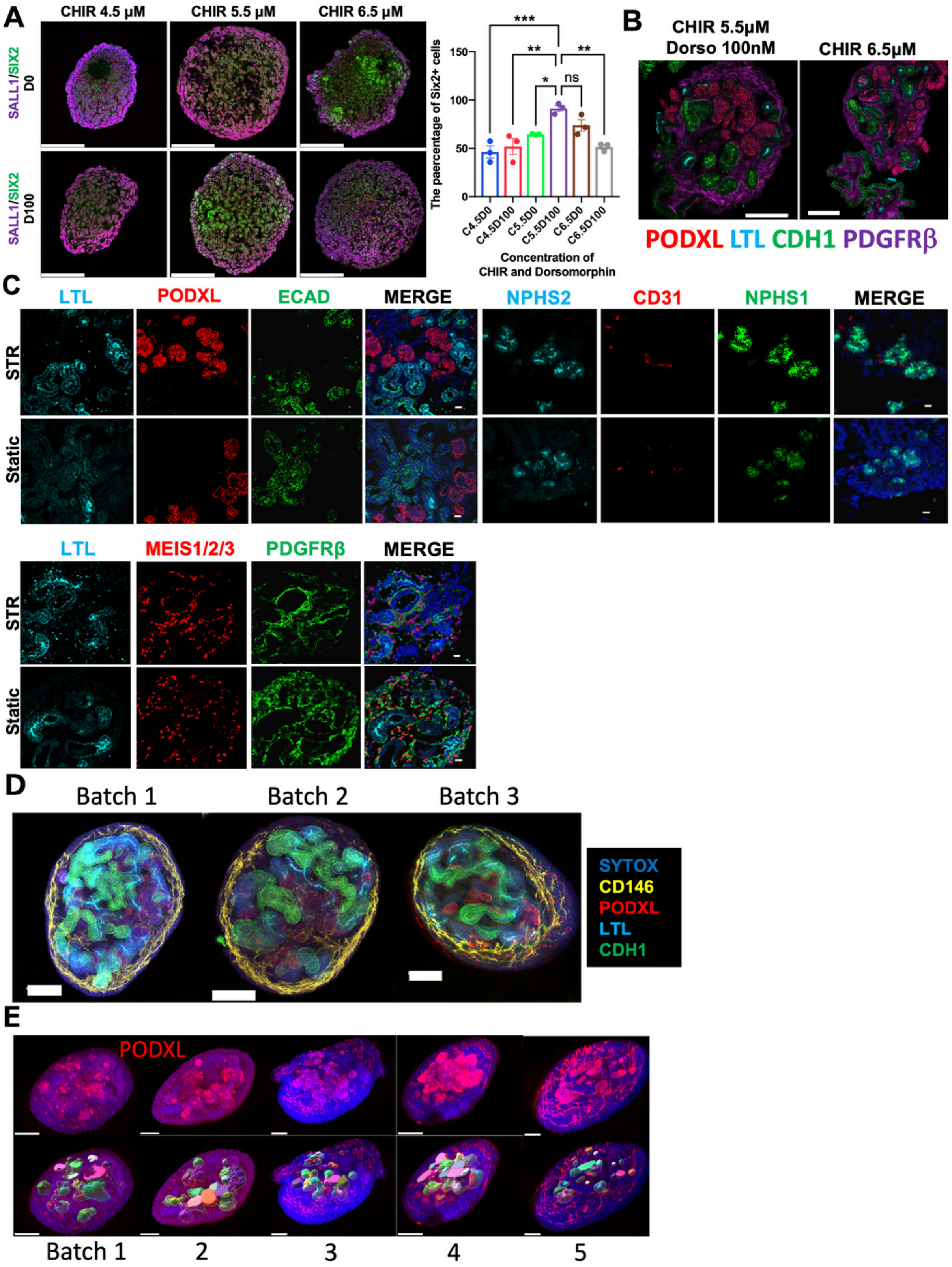
A
CHIR 4.5 μM
CHIR 5.5 μM
CHIR 6.5 μM
SALL1/SIX2 D0
SALL1/SIX2 D100
The paercentage of Six2+ cells
Concentration of CHIR and Dorsomorphin
C4.5D0
C4.5D100
C5.5D0
C5.5D100
C6.5D0
C6.5D100
***
**
*
ns
B
CHIR 5.5μM Dorso 100nM
CHIR 6.5μM
PODXL LTL CDH1 PDGFRβ
C
LTL
PODXL
ECAD
MERGE
NPHS2
CD31
NPHS1
STR
Static
MEIS1/2/3
PDGFRβ
D
Batch 1
Batch 2
Batch 3
SYTOX
CD146
PODXL
LTL
CDH1
E
PODXL
Batch 1
2
3
4
5

Supplementary Figure 3. Optimization of differentiation conditions in BJFF.6 in comparison to static organoids

(A) Confocal images of cryosectioned day 8 STR organoids differentiated at the concentrations of 4.5, 5.5, or 6.5 μM CHIR with (D100) or without (D0) 100 nM Dorsomorphin. The scale bars represent 100 μm. The graph displays SIX-2 expression by day 8 NPCs. n=3 organoids in each condition. Asterisks in the bar graphs indicate p values derived from one-way ANOVA with Tukey's multiple comparisons test. Means ± SEM. (*p ≤ 0.05, **p ≤ 0.01, ***p ≤ 0.001); ns, not significant. (B) Confocal images of cryosectioned day 21 STR organoids differentiated with either 6.5 μM CHIR alone or 5.5 μM CHIR in the presence of 100 nM Dorsomorphin. Podocytes (PODXL), proximal tubules (LTL), distal tubules (CDH1), and stromal cells (PDGFRβ). The scale bars represent 100 μm. (C) Confocal images of cryosectioned day 21 STR and static organoids differentiated at their final optimal concentration of 5.5μM CHIR+100nM Dorsomorphin (STR) or 5.5μM CHIR+100nM Dorsomorphin (static). Podocytes (PODXL, NPHS2, NPHS1), distal tubules (ECAD), vessels (CD31), proximal tubules (LTL), and stromal cells (MEIS1/2/3, PDGFRβ). The scale bars represent 100 μm. (D) Confocal images of day 21 STR organoids differentiated in the presence of 5.5 μM CHIR and 100nM Dorsomorphin. Vessels (CD146), podocytes (PODXL), proximal tubules (LTL), and distal tubules (CDH1). The scale bars represent 100 μm. (E) Count of podocyte clusters (glomerular surfaces) in each organoid of 5 different batches. The scale bars represent 100 μm.

A

Parameters	STR	Static
Yield (10^6 cells/vessel or plate)	41.9	3.2
Vessel/plate	1.0	1.0
StemFit 02 (ml)	12.0	1.2
aRPMI (ml)	75.0	98.0
Activin (ng)	100.0	80.0
FGF9 (ng)	350.0	440.0

B

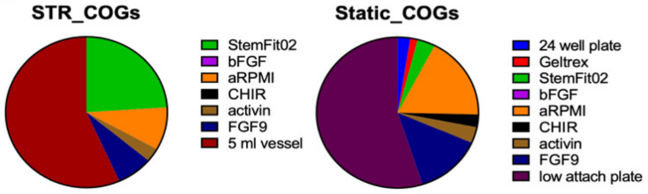

C

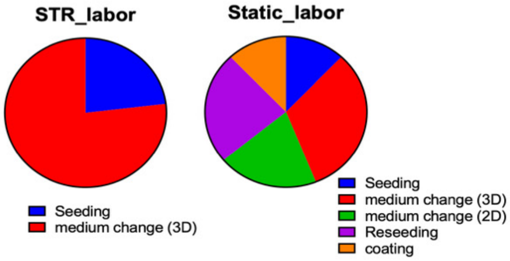

D

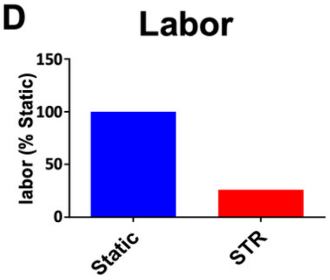

E

Parameters (h)	STR	Static
Coating	-	2.0
Seeding	1.0	2.0
Medium change	3.3	8.7
Reseeding	-	4.0

F

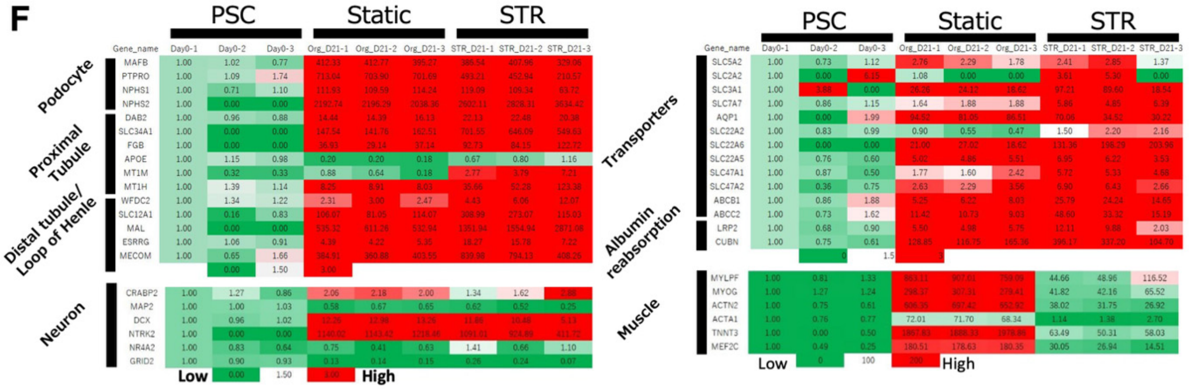

G

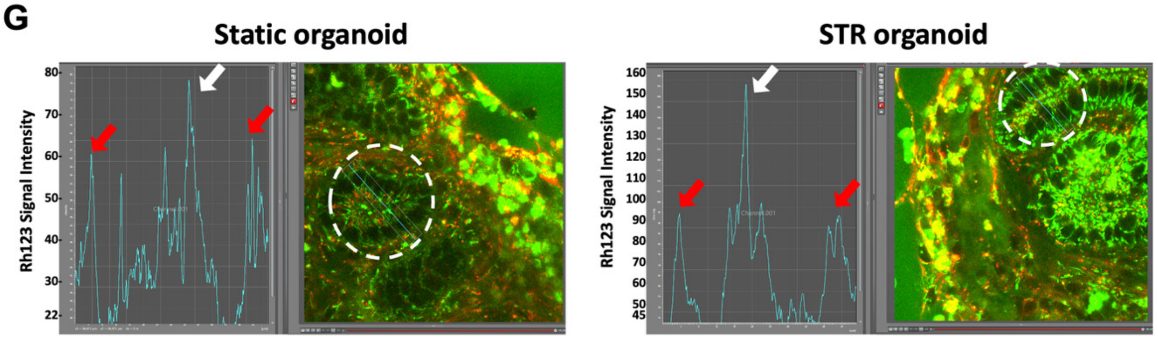

Supplementary Figure 4. Manufacturing cost and heatmap analysis of STR and static kidney organoid protocols.

(A) The comparison of STR and static kidney organoid protocols in terms of the cell yield and material consumption in one bioreactor (STR) and one plate (static). (B) The proportion of the total cost of goods/materials (COGs; cell culture reagents, media, and materials) in STR and static organoids. (C) The labor charts show the proportion of work time (cell-culture labor) spared for each experimental procedure of STR and static cultures. (D) Comparison of total work time/labor to generate static or STR organoids from 4.19 x 10^7 organoid cells. (E) The comparison of time duration (in hours) of each cell-culture labor parameter required to generate STR and static kidney organoids. (F) Comparative gene expression analysis by heatmaps of nephron segments, neurons, tubular transporters, muscles in PSCs, and day-21 organoids (static versus STR). (G) Quantification of apical and basal Rh123 signal intensities of proximal tubular epithelial cells via line analysis of tubular portions with a 10 μm width for the calculation of the apicobasal ratio. The images encircled by white dashed lines exhibit the analyzed LTL+ Rh123+ tubular portions. Red arrows indicate the signal intensities of the basal sides, while white arrows mark the signal intensities of the apical sides of the particular proximal tubules.

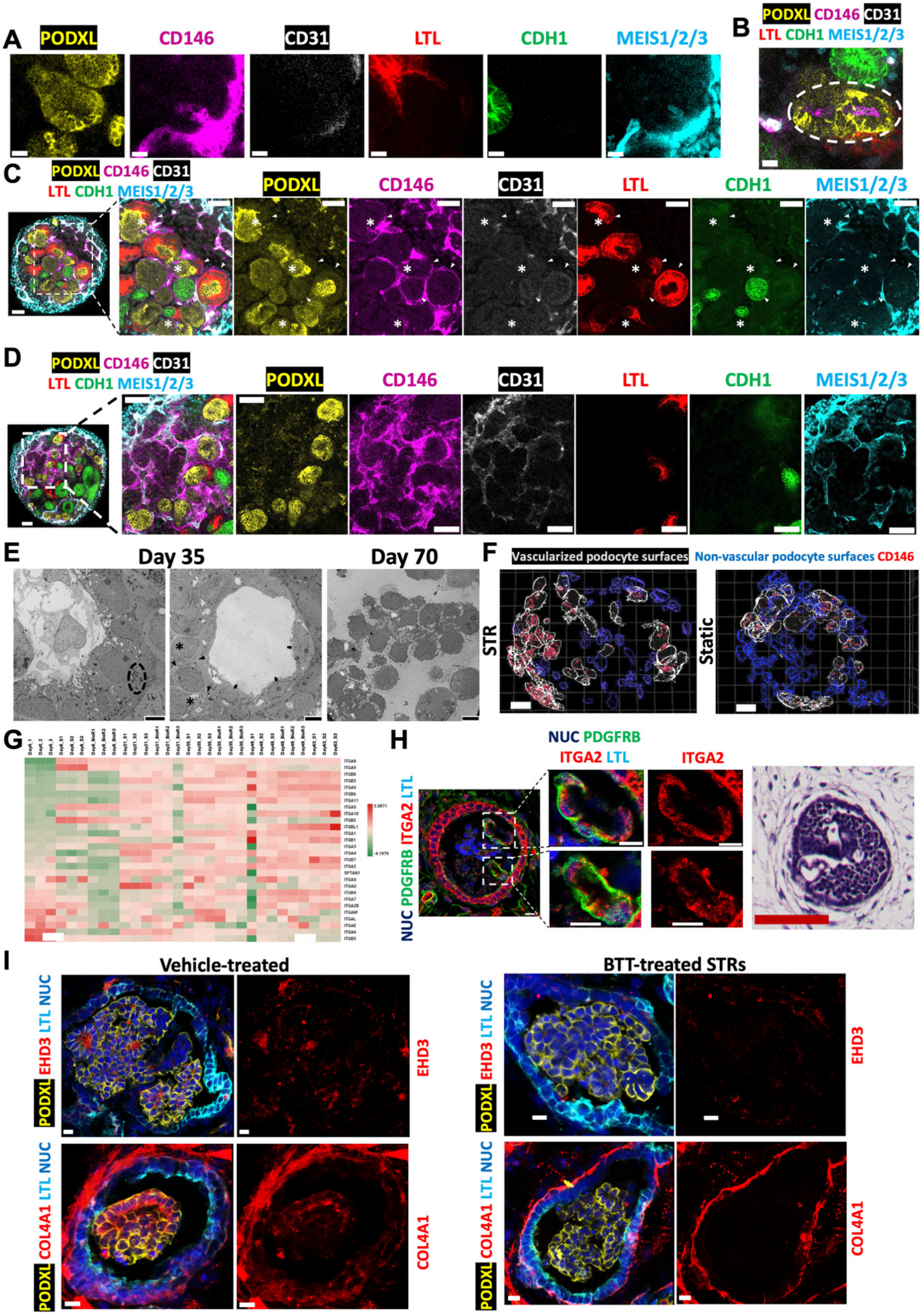
A
PODXL
CD146
CD31
LTL
CDH1
MEIS1/2/3
B
PODXL CD146 CD31
LTL CDH1 MEIS1/2/3
C
PODXL CD146 CD31
LTL CDH1 MEIS1/2/3
PODXL
CD146
CD31
LTL
CDH1
MEIS1/2/3
D
PODXL CD146 CD31
LTL CDH1 MEIS1/2/3
PODXL
CD146
CD31
LTL
CDH1
MEIS1/2/3
E
Day 35
Day 70
F
Vascularized podocyte surfaces Non-vascular podocyte surfaces CD146
STR
Static
G
ITGA8
ITGA9
ITGB8
ITGB3
ITGAV
ITGB6
ITGA11
ITGA5
ITGA10
ITGB2
ITGBL1
ITGA1
ITGB1
ITGA3
ITGA4
ITGB7
ITGA2
SPTAN1
ITGAX
ITGAD
ITGB4
ITGA7
ITGA2B
ITGAM
ITGAL
ITGAE
ITGA6
ITGB5
3.0871
-4.1979
H
NUC PDGFRB
ITGA2 LTL
ITGA2
NUC PDGFRB ITGA2 LTL
I
Vehicle-treated
BTT-treated STRs
PODXL EHD3 LTL NUC
EHD3
PODXL COL4A1 LTL NUC
COL4A1

Supplementary Figure 5. The ultrastructural architecture and vascularization pattern of ITGA2+ EHD3+ STR glomeruli with their COL4A1+ basement membrane.

(A) Single-color high-resolution images of CD146+ glomerular endothelia connected from the interstitial vascular structures in day 21 STR organoids from Fig. 4A. The scale bars: 10 μm. (B) High-magnification image of CD146+ glomerular vessel sprouts surrounded by polarized podocytes of day 21 STR organoids. The white dashed line encircles the apically polarized podocytes surrounding the CD146+ endothelia. Scale bars: 10 μm. (C) Peritubular capillaries surrounding LTL+ proximal and CDH1+ distal tubules, accompanied by vascularized podocyte clusters in day 21 STR organoids. In single and multi-color high-resolution images, asterisks mark the vascular invasion of podocyte clusters, while arrowheads show peritubular capillaries around proximal and distal tubules. Scale bars: 50 μm. (D) Single and multi-color high-resolution images of non-vascularized podocyte clusters in day 21 static organoids. Scale bars: 50 μm. (E) Transmission electron microscopy images demonstrate the ultrastructure and metabolic fitness of STR organoids on days 35 and 70 of differentiation. A black dashed circle in the left image indicates the presence of mitochondria-rich basal portions in proximal tubular epithelial cells on day 35. The asterisks and arrowheads at the central image mark the nuclei of podocytes and their surrounding foot processes, respectively, while the arrows indicate the vascular endothelial cells in day 35 STR organoids. Black scale bars: 4 μm. (F) Representative images of day 35 STR and static organoids differentiated from HUES62 cells. These STR organoids were transferred from 96-well plates into bioreactors on day 14 of differentiation. White podocyte surfaces show CD146+ vascularized podocyte clusters (red), while blue surfaces represent non-vascularized podocyte clusters. Scale bars: 100 μm. (G) The Heatmap showing the time-course differential expression of integrin α (ITGA) and β (ITGB) subtypes by static (S) and STR (BioR) organoids. (H) ITGA2 expression by PDGFR-β (PDGFRB)+ mesangial cells, which surround the glomerular capillary-like structures in STR organoids. Paraffin sections of a self-assembled nephron sheet on day 56 (prepared from day 14 STR organoids) were used for the immunostaining of ITGA2. Scale bars of immunofluorescence images represent 10 μm. Hematoxylin-eosin (HE) staining of the same nephron sheet reveals the glomerular capillary-like structures. The scale bar of the HE-stained section represents 100 μm. (I) Immunostaining for EHD3+ glomerular endothelial cells and COL4A1+ (Collagen IV α1) basement membrane in the control or BTT3033-treated STR organoids. Frozen sections of vehicle- and BTT3033-treated STR organoids (1 μM for 18 days) were stained with the designated markers. Anti-EHD3 and anti-COL4A1 were separately applied as they were both rabbit-derived antibodies. Scale bars represent 10 μm.

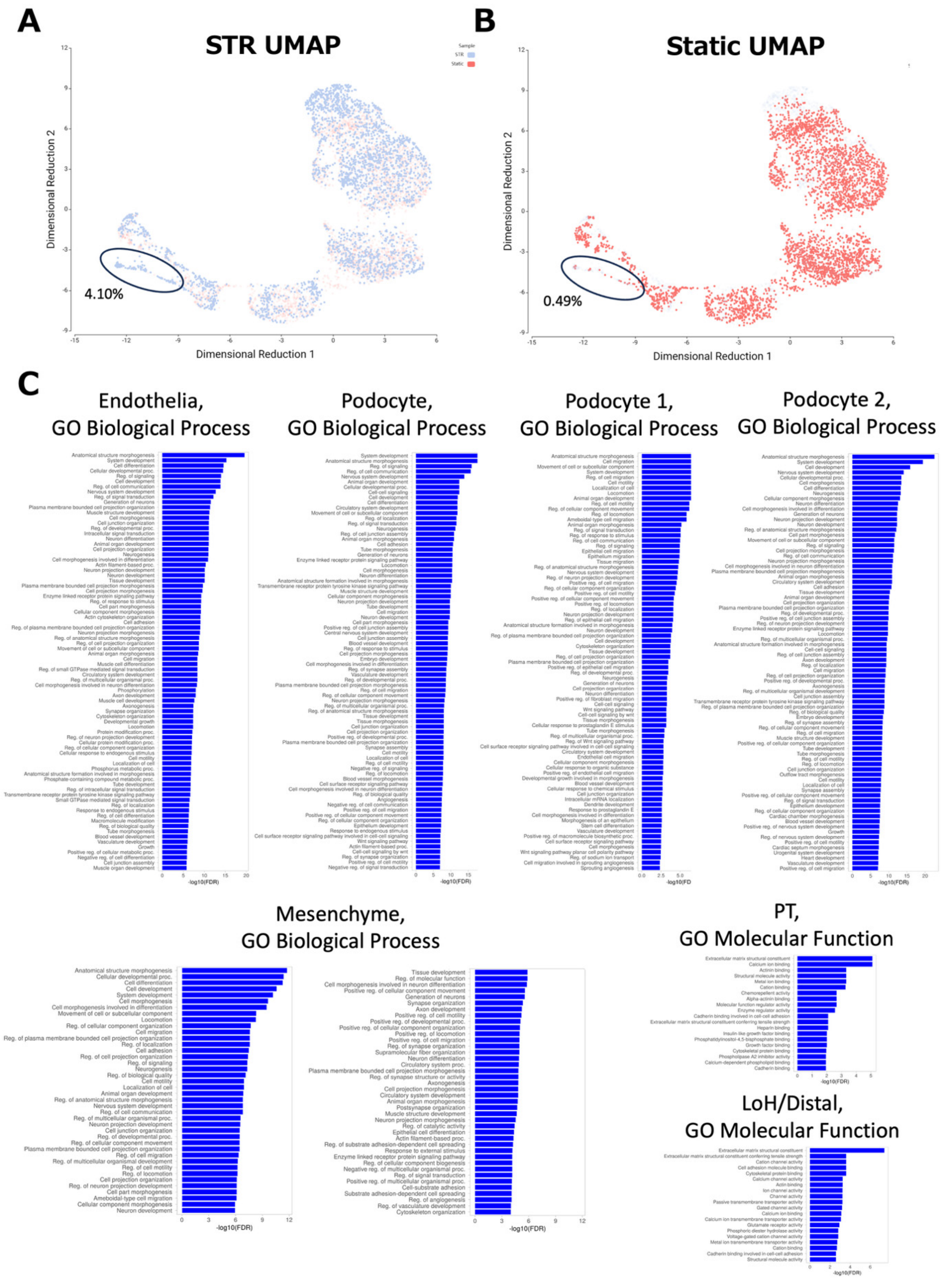
A
STR UMAP
Sample
STR
Static
Dimensional Reduction 2
Dimensional Reduction 1
4.10%
B
Static UMAP
Dimensional Reduction 2
Dimensional Reduction 1
0.49%
C
Endothelia,
GO Biological Process
Podocyte,
GO Biological Process
Podocyte 1,
GO Biological Process
Podocyte 2,
GO Biological Process
Mesenchyme,
GO Biological Process
PT,
GO Molecular Function
LoH/Distal,
GO Molecular Function
-log10(FDR)

Supplementary Figure 6. UMAP and GO term analysis of STR versus static organoids by single-cell RNA-sequencing.

(A, B) Uniform Manifold Approximation and Projections (UMAPs) represent the STR (A) and static organoids (B). The black circles: Endothelia 3 cluster. The percentage shown in the figures represents the cell percentage of Endothelia 3 cluster against total cell numbers in each condition, STR or static. (C) Bar graphs show the representative GO terms of biological processes in the clusters of Endothelia, Mesenchyme, Podocyte, and its Podocyte 1 and 2 subsets, together with molecular functions in the clusters of PT and LoH/Distal, significantly associated with upregulated DEGs in STR organoids compared to static ones.

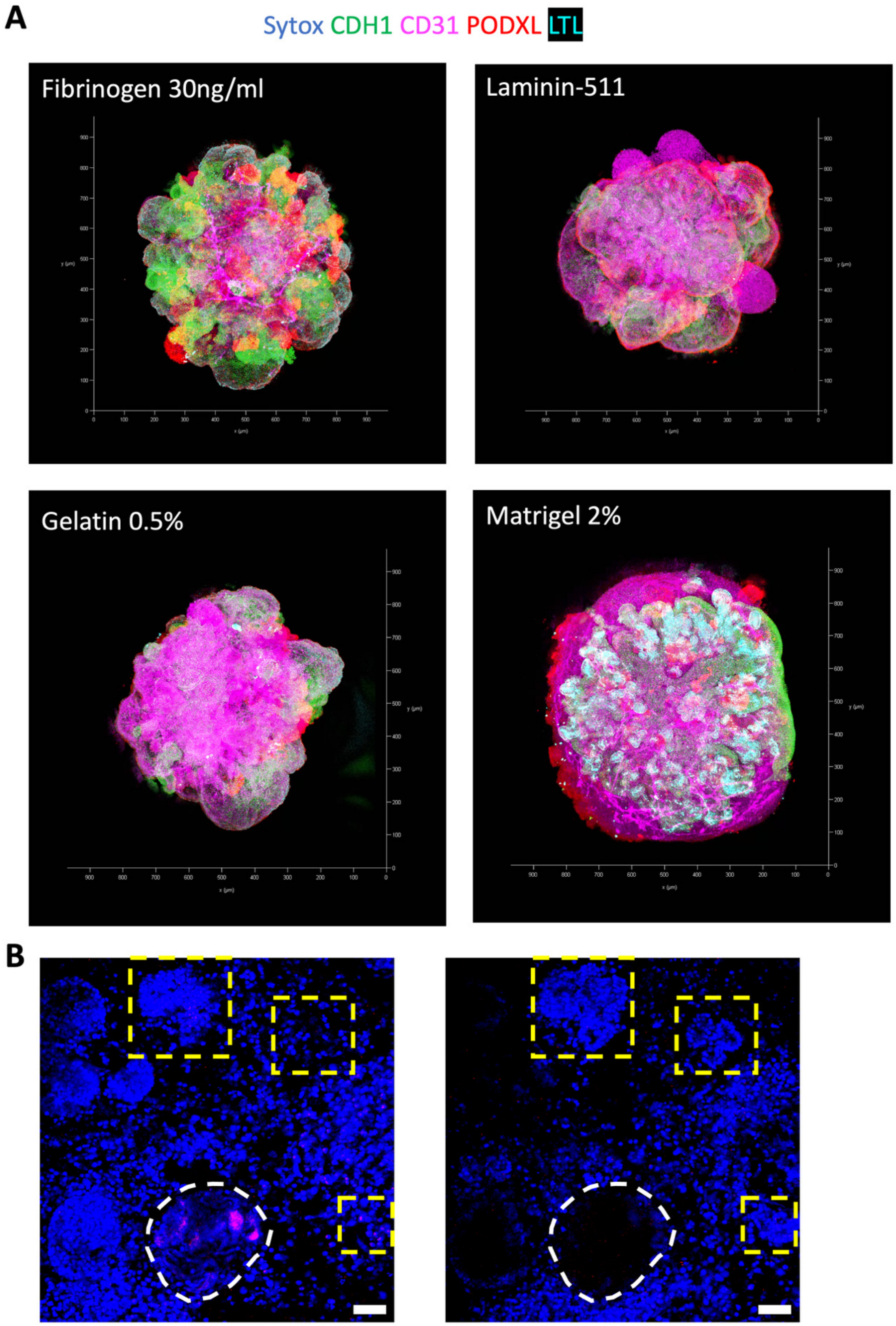

A
Sytox CDH1 CD31 PODXL LTL
Fibrinogen 30ng/ml
Laminin-511
Gelatin 0.5%
Matrigel 2%
B

Supplementary Figure 7. Effects of ECMs on nephron differentiation and the gating strategy of structures in intravital imaging.

(A) Day 14 nephron organoid shows various segmentations after being exposed to different types of ECMs. ECMs in general induced the development of the epithelial (proximal and distal) part of the nephrons. Cystic tubules were observed in organoids that were exposed to Fibrinogen (mostly CDH1+distal part), laminin-511, and Gelatin. However, Matrigel did not show an apparent impact on nephron differentiation. n=4 organoids derived from H9 & BJFF.6. (B) Phase 3 multiphoton intravital microscopy (MP-IVM) captures Hoechst+ clusters at the different Z-stack images of the implanted sheet, revealing the glomerular structures (designated by a circular white dashed line) containing the vascular components with dynamic flow of high-molecular-weight (HMW, 500 kDa-Cy5) dextran and tubular structures (designated by rectangular yellow dashed lines). Glomerular and tubular structures are recognized in left and right Z-stack images, respectively. The scale bar displays 50 μm.

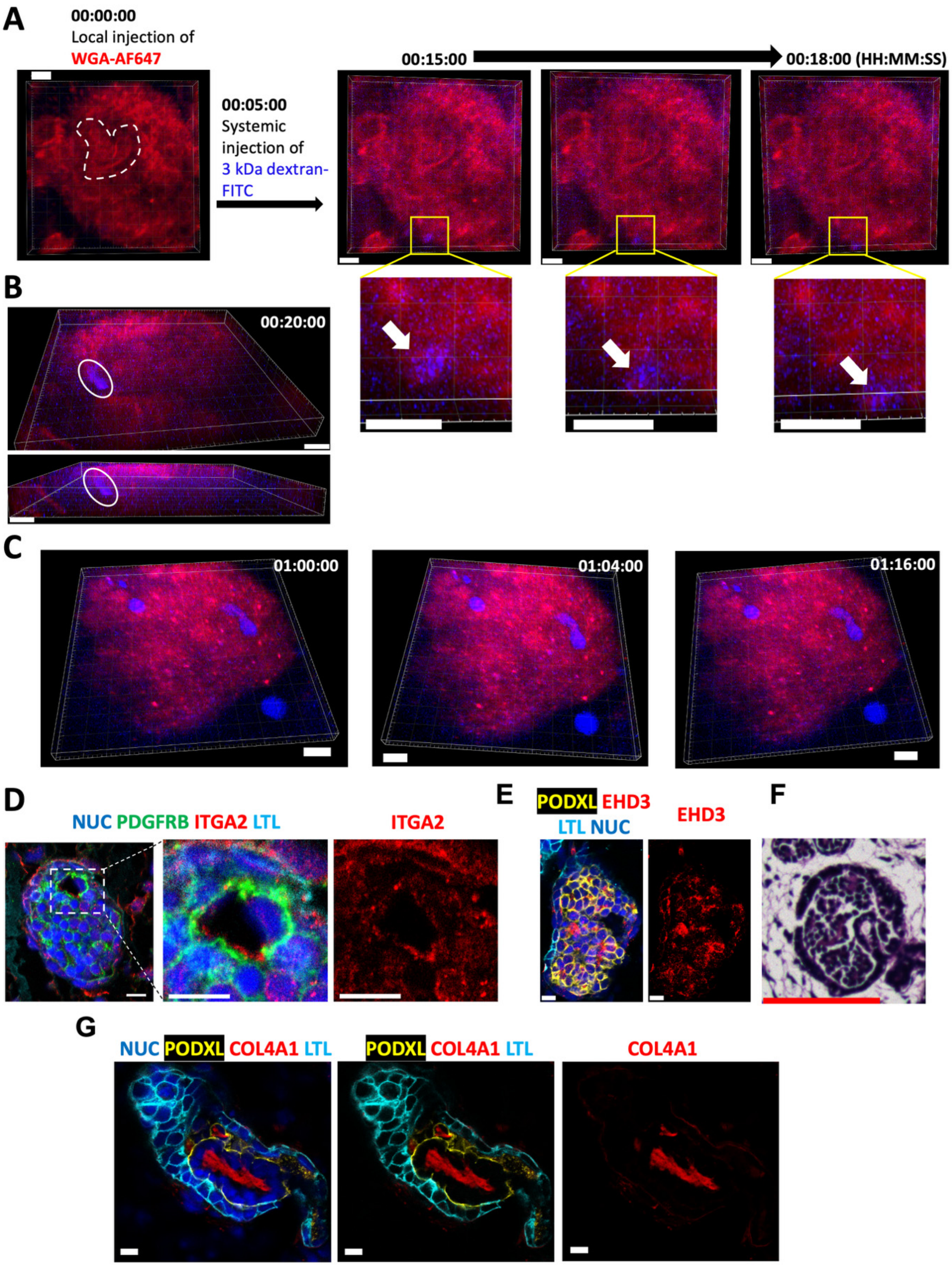

A
00:00:00
Local injection of
WGA-AF647
00:05:00
Systemic
injection of
3 kDa dextran-
FITC
00:15:00
00:18:00 (HH:MM:SS)
B
00:20:00
C
01:00:00
01:04:00
01:16:00
D
NUC PDGFRB ITGA2 LTL
ITGA2
E
PODXL EHD3
LTL NUC
EHD3
F
G
NUC PODXL COL4A1 LTL
PODXL COL4A1 LTL
COL4A1

Supplementary Figure 8. Intravital imaging of implanted nephron sheets and post-fixed tissue.

(A) The active flow of 3 kDa dextran in implanted nephron sheets (6 days after the implantation) detected by multiphoton intravital imaging (MP-IVM). Conjugated WGA with Alexa Fluor 647 (AF647, red) was locally injected into the nephron sheet in the murine DSFC. Next, 3 kilodalton (kDa) low-molecular-weight (LMW) dextran conjugated with fluorescein (FITC, blue) was systemically administered to mice. The nephron sheet was visualized by MP-IVM at the indicated time points. Below are the zoomed-in images. (B) Accumulation of 3 kDa dextran in the tubular structure of the same nephron sheet shown at different perspectives 15 min after the injection of LMW dextran. (C) Long-term collection of 3 kDa dextran in the tubular structures and/or interstitial spaces within the nephron sheet at indicated time points after the local and systemic injection of WGA-AF67 and 3 kDa dextran-FITC, respectively. Scale bars in (A), (B), and (C): 70 μm. (D) ITGA2 expression in PDGFRB+ cells surrounding the glomerular capillary-like structures in the implanted nephron sheet. After the nephron sheet in (A-C) was explanted from mice at the end of MP-IVM, they were fixed to obtain paraffin and frozen sections for the immunostaining and subsequent imaging by confocal microscopy. Paraffin sections were used for ITGA2 staining. Scale bars: 10 μm. (E) Preservation of EHD3+ glomerular endothelial cells in the implanted nephron sheet. Frozen sections of the explanted nephron sheet in (A-C) were used for EHD3 staining. Scale bars: 10 μm. (F) Hematoxylin-eosin (HE) staining of the same nephron sheet reveals the glomerular capillary-like structures. The scale bar represents 100 μm. (G) Detection of COL4A1+ basement membrane twining the human glomerular capillary network. Frozen sections of the nephron sheet in (A-C) were also used for COL4A1 staining. Anti-EHD3 and anti-COL4A1 were separately applied as they were both rabbit-derived antibodies. Scale bars: 10 μm.

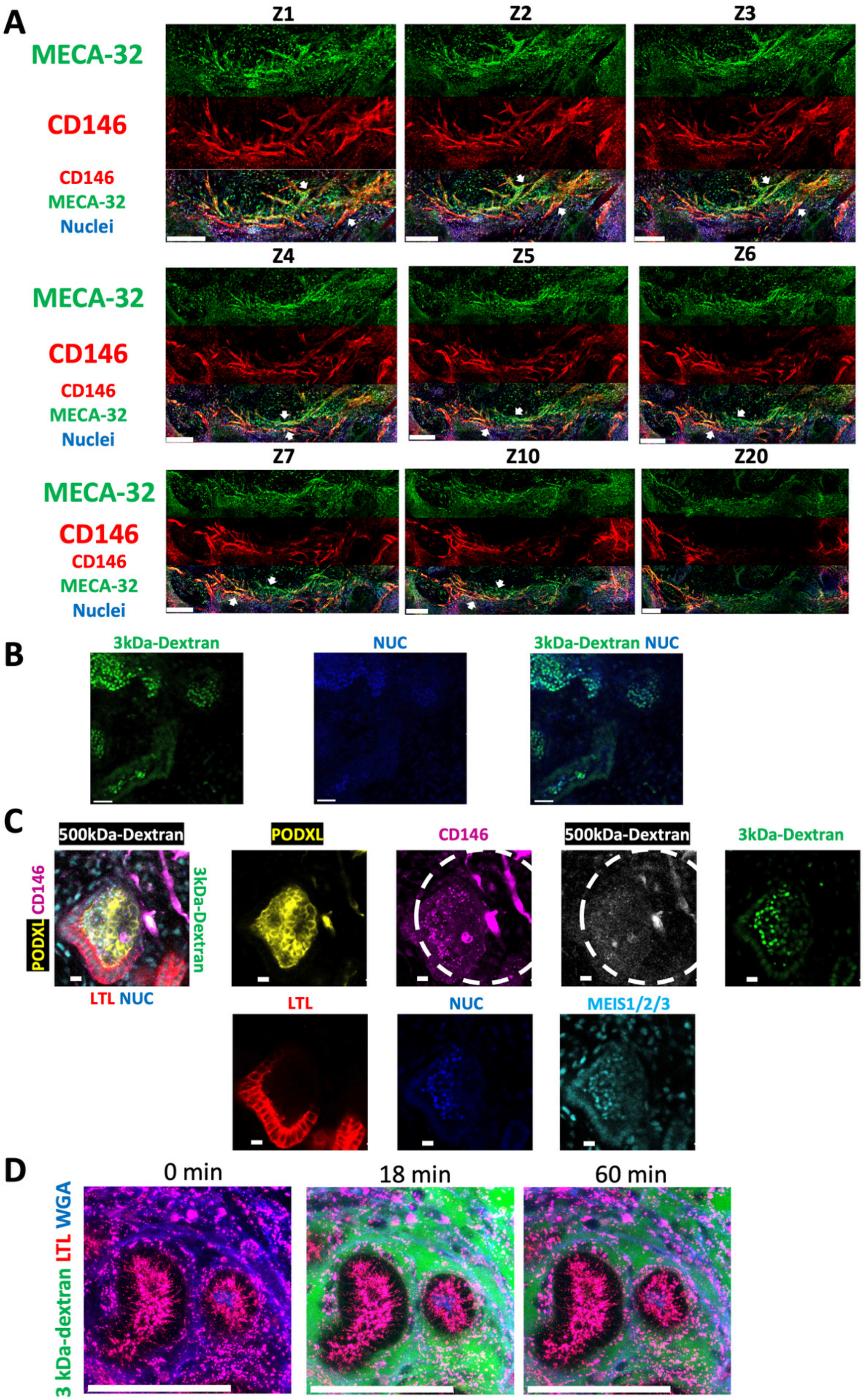

A
Z1
Z2
Z3
MECA-32
CD146
CD146
MECA-32
Nuclei
Z4
Z5
Z6
MECA-32
CD146
CD146
MECA-32
Nuclei
Z7
Z10
Z20
MECA-32
CD146
CD146
MECA-32
Nuclei
B
3kDa-Dextran
NUC
3kDa-Dextran NUC
C
500kDa-Dextran
PODXL CD146
3kDa-Dextran
LTL NUC
PODXL
CD146
500kDa-Dextran
3kDa-Dextran
LTL
NUC
MEIS1/2/3
D
0 min
18 min
60 min
3 kDa-dextran LTL WGA

Supplementary Figure 9. Post-fixed images in implanted nephron sheets and live-cell imaging of STR organoids *in vitro*.

(A) The confocal microscopy images at different Z stack positions reveal the anastomosis of CD146+ human and MECA-32+ murine vascular endothelial networks. The interval between each Z position is 5 μm. The white arrows show the interacting murine (MECA-32, green) and human (CD146, red) vascular endothelial network. Scale bars: 150 μm. (B) 3 kDa-dextran and nuclear single-channel images of the fixed implanted nephron sheet in Figure 7D. (C) Differential localization of HMW (500 kDa) and LMW (3 kDa) dextrans in the vascularized glomeruli of the implanted nephron sheet. A circular white dashed line shows the localization of HMW dextran inside CD146+ glomerular and extra-glomerular vessels. Scale bars: 10 μm. (D) In vitro application of LMW dextran demonstrated no uptake of LMW dextran by proximal tubular cells of day 40 STR organoids. Proximal tubules of STR organoids in chamber slides were labeled with Wheat Germ Agglutinin (WGA)-Alexa Fluor 555 (WGA-AF555) and LTL-Alexa Fluor 647 at 37°C overnight at dilution ratios of 1:200 and 1:100, respectively. The next day, LTL-labeled STR organoids were visualized via live-cell imaging by confocal microscopy. The “0 min” image represents the baseline image just before the addition of LMW dextran into the organoid culture medium. Following the supplementation of LMW dextran at a final concentration of 10 μg/ml (1:1000 dilution), the time-lapse imaging was performed for 1 hour. Scale bars: 100 μm.

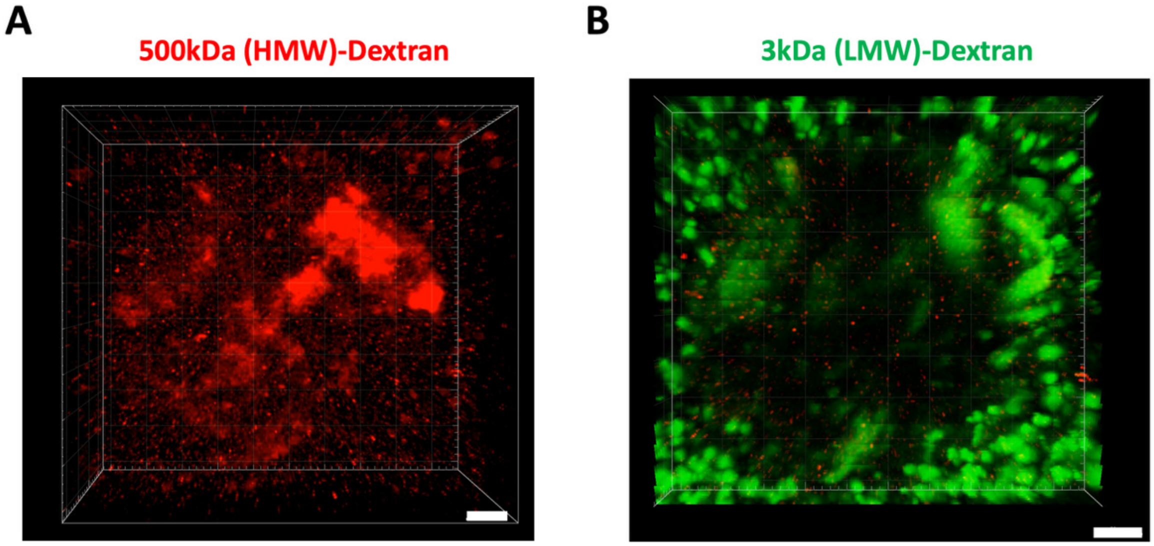
A
500kDa (HMW)-Dextran
B
3kDa (LMW)-Dextran

Supplementary Movie 1. Selective filtration of 3 kDa dextran compared to 500 kDa dextran. The movie of each channel for 500 kDa-Cy5 (high-molecular-weight (HMW)) in (A) and 3 kDa-FITC (low-molecular-weight (LMW)) dextrans in (B) reflects the dynamic process of filtration. The scale bars represent 20 μm.

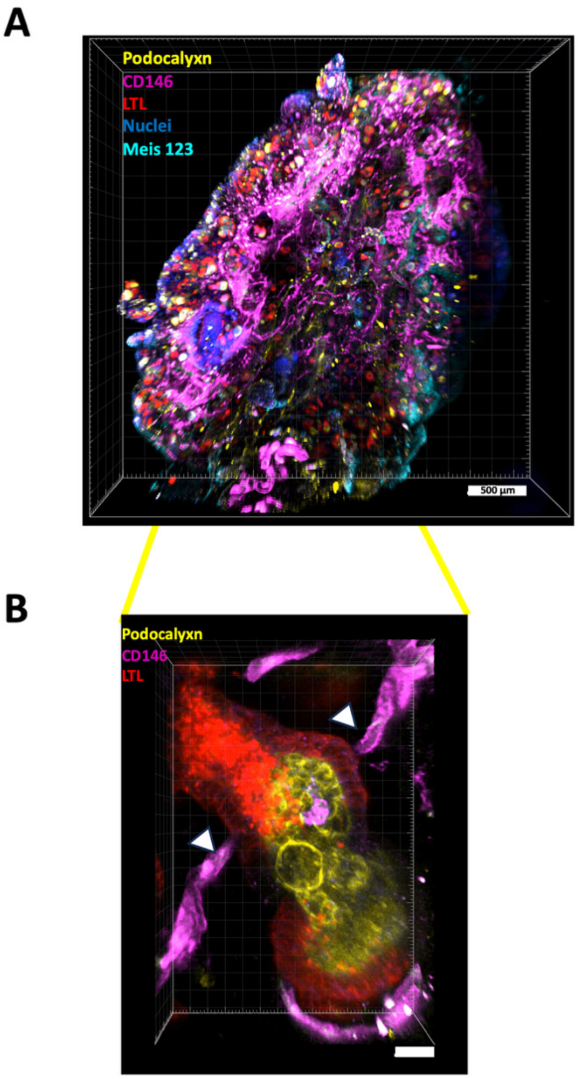

Vascularized glomeruli in the explanted nephron sheet

Supplementary Movie 2. Human glomerular vascularization inside the explanted nephron sheet from murine DSFC. 3D movies exhibit the persistence of human glomerular vascularization. (A) represents the explanted nephron sheet from the murine DSFC, while (B) shows the zoomed-in vascularized glomeruli of this sheet. The scale bars in (A) and (B) display 500 µm and 20 µm, respectively.
